# Single‐Cell Analysis Reveals Malignant Cells Reshape the Cellular Landscape and Foster an Immunosuppressive Microenvironment of Extranodal NK/T‐Cell Lymphoma

**DOI:** 10.1002/advs.202303913

**Published:** 2023-11-10

**Authors:** Yi‐Qi Li, Chun‐Ling Luo, Jia‐Xin Jiang, Shuai He, Yang Liu, Wen‐Xin Yan, Yi Xia, Qian Cui, Ying Huang, Jing Quan Lim, Dachuan Huang, Izzah Nabilah Hussein, Yan Gao, Guo‐Wang Lin, Yi‐Hong Ling, Dong Ma, Yue‐Tong Zhang, Jason Yongsheng Chan, Pan‐Pan Wei, Xiao‐Xiao Wang, Chee Leong Cheng, Jie Xiong, Wei‐Li Zhao, Choon Kiat Ong, Soon Thye Lim, Hui‐Qiang Huang, Rou‐Jun Peng, Jin‐Xin Bei

**Affiliations:** ^1^ State Key Laboratory of Oncology in South China Guangdong Provincial Clinical Research Center for Cancer Sun Yat‐sen University Cancer Center Guangzhou 510060 China; ^2^ Guangdong Provincial People's Hospital Guangdong Academy of Medical Sciences Guangzhou 510080 China; ^3^ Lymphoma Translational Research Laboratory Cellular and Molecular Research National Cancer Centre Singapore 30 Hospital Boulevard Singapore 168583 Singapore; ^4^ ONCO‐ACP Duke‐NUS Medical School 8 College Road Singapore 169857 Singapore; ^5^ Microbiome Medicine Center Division of Laboratory Medicine Zhujiang Hospital Southern Medical University Guangzhou 510280 China; ^6^ Division of Medical Oncology National Cancer Centre Singapore 30 Hospital Boulevard Singapore 168583 Singapore; ^7^ Department of Pathology Singapore General Hospital 20 College Road Academia 169856 Singapore; ^8^ State Key Laboratory of Medical Genomics Shanghai Institute of Hematology National Research Center for Translational Medicine Shanghai Rui Jin Hospital Shanghai Jiao Tong University School of Medicine 197 Rui Jin Er Road Shanghai 200025 China; ^9^ Cancer and Stem Cell Biology Duke‐NUS Medical School 8 College Road Singapore 169857 Singapore; ^10^ Director's Office National Cancer Centre Singapore 30 Hospital Boulevard Singapore 168583 Singapore; ^11^ Office of Education Duke‐NUS Medical School Singapore 169857 Singapore

**Keywords:** DPP4, immunosuppression, LMP1^+^ malignant NK cells, NKTCL, scRNA‐seq

## Abstract

Extranodal natural killer/T‐cell lymphoma (NKTCL) is an aggressive type of lymphoma associated with Epstein–Barr virus (EBV) and characterized by heterogeneous tumor behaviors. To better understand the origins of the heterogeneity, this study utilizes single‐cell RNA sequencing (scRNA‐seq) analysis to profile the tumor microenvironment (TME) of NKTCL at the single‐cell level. Together with in vitro and in vivo models, the study identifies a subset of LMP1^+^ malignant NK cells contributing to the tumorigenesis and development of heterogeneous malignant cells in NKTCL. Furthermore, malignant NK cells interact with various immunocytes via chemokines and their receptors, secrete substantial DPP4 that impairs the chemotaxis of immunocytes and regulates their infiltration. They also exhibit an immunosuppressive effect on T cells, which is further boosted by LMP1. Moreover, high transcription of EBV‐encoded genes and low infiltration of tumor‐associated macrophages (TAMs) are favorable prognostic indicators for NKTCL in multiple patient cohorts. This study for the first time deciphers the heterogeneous composition of NKTCL TME at single‐cell resolution, highlighting the crucial role of malignant NK cells with EBV‐encoded LMP1 in reshaping the cellular landscape and fostering an immunosuppressive microenvironment. These findings provide insights into understanding the pathogenic mechanisms of NKTCL and developing novel therapeutic strategies against NKTCL.

## Graphical abstract

Single‐cell RNA‐sequencing analysis and functional investigations uncover the pivotal role of EBV, particularly the viral gene LMP1, in the malignant transformation and progression of NKTCL. Malignant NK cells entertain with immune cells to jointly foster an immunosuppressive TME favorable for NKTCL development. Targeting TME components, including LMP1 and DPP4, emerges as a potential therapeutic strategy against NKTCL.

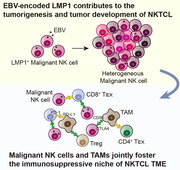

## 1 Introduction

Extranodal natural killer/T‐cell lymphoma (NKTCL) is a highly aggressive lymphoma associated with Epstein–Barr virus (EBV) infection and predominantly occurs in Asian and Latin American populations.^[^ ^1^ ^]^ Various chemotherapeutic approaches including P‐GEMOX,^[^ ^2^ ^]^ DeVIC,^[^ ^3^ ^]^ VIDL,^[^ ^4^ ^]^ SMILE regimens,^[^ ^5^ ^]^ and their combination with concurrent radiotherapy are widely practiced strategies for NKTCL.^[^ ^6^ ^]^ Nevertheless, the overall survival of patients with NKTCL is poor, and even more dismal for those with relapsed or refractory NKTCL, with average survival of only 6.4 months.^[^ ^7^, ^8^ ^]^ Most recently, novel therapeutic regimes such as immune checkpoint inhibitors and histone deacetylase inhibitors have demonstrated beneficial outcomes for advanced or relapsed/refractory NKTCL, but only for a limited proportion of patients.^[^ ^9^, ^10^, ^11^, ^12^, ^13^, ^14^ ^]^ These varied behaviors and treatment responses highlight the intrinsic heterogeneity of NKTCL.

Pathologically, NKTCL is NK or T cell origin and exhibits pleomorphic cell morphology with small or medium‐sized cells to large transformed cells.^[^ ^15^, ^16^, ^17^ ^]^ At molecular aspects, our previous studies have identified genetic variants, including *HLA‐II* and *IL18RAP*, conferring variable susceptibility of NKTCL.^[^ ^18^, ^19^ ^]^ Furthermore, considering that EBV variations (like type 1 and type 2 strains) may contribute to different transforming abilities,^[^ ^20^, ^21^ ^]^ we have recently demonstrated that EBV alterations, including specific mutations, host integrations, and abnormal expression of viral genes, vary among NKTCL tumors.^[^ ^22^ ^]^ Moreover, genomic and transcriptomic sequencing studies have revealed the accumulation of somatic mutations and dysregulation of gene expression in specific NKTCL samples, providing valuable information for molecular subtyping of patients.^[^ ^15^, ^23^ ^]^ Together, these multidimensional factors foster tumor heterogeneity of NKTCL, although they only represent a few aspects of the overall cancer hallmarks.

Apart from heterogeneous cancer cells, substantial normal cells in multiple cancers jointly establish tumor microenvironment (TME) and contribute to another dimension of heterogeneity for the maintenance of cancer hallmarks.^[^ ^24^ ^]^ Single‐cell transcriptome analyses have revealed heterogeneous immune cells and stromal cells in the TME of multiple cancers, highlighting the importance of specific cell subtypes and cellular interactions in tumor progression and treatment responses.^[^ ^25^, ^26^, ^27^ ^]^ Similarly, intensive infiltration of lymphocytes is one of the major histopathological characteristics of NKTCL.^[^ ^28^ ^]^ However, the cellular architecture of NKTCL TME and its potential association with NKTCL progression remain unclear. Here, we aimed to decipher the heterogeneous TME of NKTCL, identify key cell types and cellular interactions associated with mechanisms underlying NKTCL progression through single‐cell transcriptome analyses and functional investigations. These findings provide valuable insights into the development of precise therapies targeting NKTCL.

## 2 Results

### 2.1 Single‐Cell Landscape of Tumor Microenvironment (TME) in Patients with Extranodal Natural Killer/T‐Cell Lymphoma (NKTCL)

**Figure 1:**
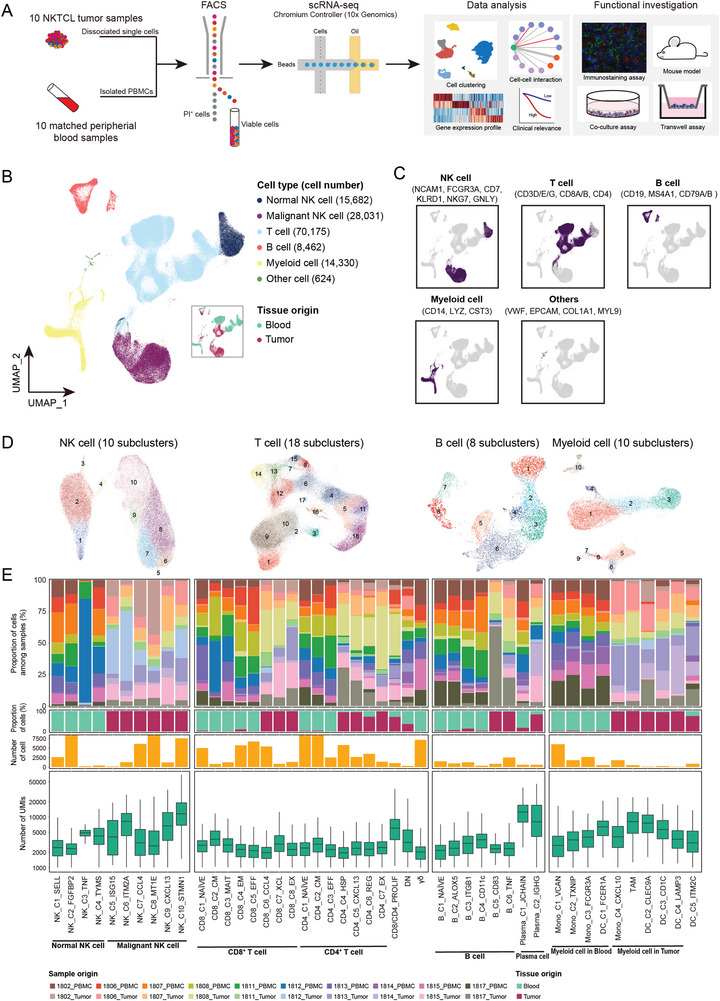
Landscape profiling of NKTCL TME at single‐cell resolution. A) An overview of the study design. Single viable cells from matched tumor and peripheral blood samples were collected using fluorescence‐activated cell sorting (FACS) and subjected for cell barcoding. The cDNA libraries of 5′‐mRNA expression were constructed, followed by high throughput sequencing, downstream data analyses, and functional validations of key findings. B) UMAP plot showing a total of 137 304 cells clustered into six major cell types. Each dot represents a cell, colored according to its cell type as indicated at the right panel. The inlet plot shows the tissue distribution of cells colored according to their origins from either peripheral blood or tumor. C) Expression of canonical marker genes to define the major cell type on top. Each dot represents a cell, and the depth of color from light grey to deep purple represents low to high expression of marker genes. D) UMAP plots showing the subclusters of NK, T, B, and myeloid cells. Each dot represents a cell, colored according to its cell subtype. E) Diagrams showing basic information of each subcluster for NK, T, B, and myeloid cells (panels from top to bottom): the proportions of cells derived from ten patients with corresponding colors indicated at the bottom, the proportions of cells from either peripheral blood (cyan) or tumor (dark red), the numbers of cells, and the box plots of the numbers of UMIs. For box plots, center lines and whiskers denote median values and 1.5× the interquartile range, respectively.

To delineate the heterogeneous cell composition of NKTCL, we performed single‐cell RNA sequencing (scRNA‐seq) on viable cells derived from 10 treatment‐naïve NKTCL tumors and their matched peripheral blood mononuclear cells (PBMCs; **Figure** **1**A; Figure S1A and Table S1, Supporting Information). After stringent quality control (Figure S1B,C, Supporting Information), we identified a total of 137 304 cells from all samples in the scRNA‐seq cohort, including 58 057 cells from tumor and 79 247 cells from peripheral blood (Table S2a, Supporting Information). We defined cell types for each cell cluster according to canonical markers, among which four major cell types were identified, including NK, T, B, and myeloid cells (Figure 1B,C). Using inferCNV with our reference dataset,^[^ ^29^ ^]^ we identified a high degree of chromosomal copy number variations (CNVs) in NK cells for all tumor samples (Figure S2A, Supporting Information), indicating their NK‐cell origin. In addition, we observed the expression of histologically diagnostic markers, including EBV‐encoded genes and cytotoxic molecules, in malignant NK cells (Figure S2B–D, Supporting Information), consistent with the EBV‐related nature and pathological characteristics of NKTCL.^[^ ^1^ ^]^

We further identified multiple subclusters within each major cell type, showing both intraindividual and interindividual variabilities in cell proportions (Figure 1D,E, Figure S3A and Table S2b, Supporting Information). Immune cell clusters were found in both tumor and peripheral blood, in contrast to the in situ location of malignant NK cells (Figure 1E, Table S2, Supporting Information), indicating a common infiltration of immune cells in NKTCL as other cancers (Figure S4A, Supporting Information). Moreover, we observed a decrease in the proportions of normal NK, T, and myeloid cells in tumors compared with blood samples, suggesting two distinct immune landscapes between tumor and peripheral blood in NKTCL (Figure S4B, Supporting Information). Intriguingly, we detected a tiny proportion of normal NK cells in NKTCL tumors (0.32%), significantly lower than other cancers (Figure S4C, Supporting Information). Additionally, we identified exhausted and regulatory T cells, as well as pro‐tumorigenic tumor‐associated macrophages (TAMs), according to their canonical markers, which distributed predominantly in tumors (Figure S5 and Table S3, Supporting Information).

### 2.2 Heterogeneous Characteristics of Malignant NK Cells in NKTCL

**Figure 2:**
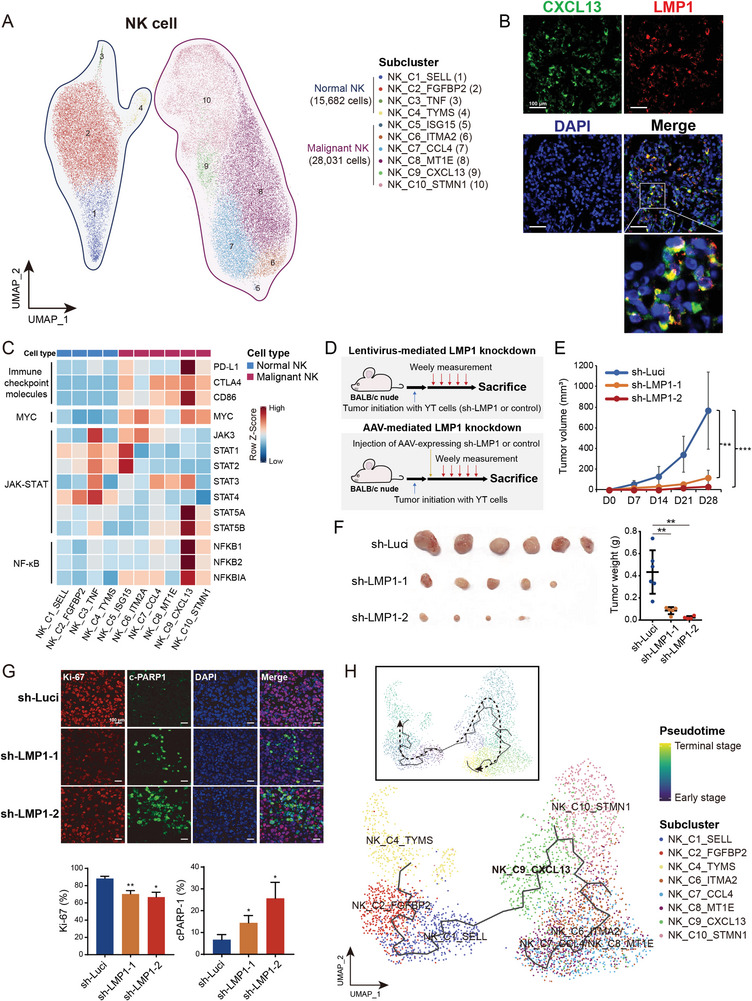
The pivotal role of LMP1^+^ NK_C9_CXCL13 cells in oncogenic activation and development of NKTCL. A) UMAP plot showing 43713 NK cells grouped into ten clusters, including four normal clusters and six malignant clusters. Each dot represents a cell, colored according to its cell cluster as indicated at the right panel. B) Multiplex IF staining for NK_C9_CXCL13 malignant NK cells (CXCL13^+^LMP1^+^) in NKTCL biopsies from the SC‐cohort. CXCL13 and LMP1 proteins as well as nuclear DNA are detected with different colors as indicated on top. Images are representative of biological replicates from three patients. C) Heatmap showing the expression levels of selected genes (rows) for each NK cell cluster (columns). NK clusters of either normal (blue) or malignant NK (purple) are indicated as rectangles on top. Filled colors from blue to red in the squares represent normalized expression levels from low to high as scaled in row direction (row Z‐score). D) Experimental design for the in vivo blockade of LMP1 in BALB/c nude mice. For lentivirus‐mediated LMP1 knockdown (top panel), NKTCL mouse models were established with tumor initiation of NKTCL cells (YT) infected with sh‐LMP1 or control lentiviruses. For adeno‐associated virus (AAV)‐mediated LMP1 knockdown (bottom panel), NKTCL mouse models were established with tumor initiation of wild‐type YT cells, and intratumor injection of AAV‐expressing sh‐LMP1 or corresponding control was performed when the xenografts had grown to a certain volume. E) Tumor growth of xenografts derived from YT cells infected with lentivirus‐expressing shRNAs targeting *LMP1* (sh‐LMP1‐1/‐2) or scrambled (sh‐Luci) at different time courses (day), with the tumor size (F) left panel) and tumor weight (F) right panel) for the excised xenografts. G) Multiplex IF staining assays (top panel) for the protein expression of Ki‐67 and c‐PARP1 in malignant NK cells for the tumor section of the xenografts excised from (E) and bar plots (bottom panel) for the percentages of Ki‐67^+^/c‐PARP1^+^ cells. H) Pseudotime development trajectories of malignant and normal NK cells. Each dot represents a cell in the trajectory projection, colored according to NK cell clusters. The inlet plot shows cells colored according to predicted pseudotime scores from deep blue to yellow, representing cell states from early stage to terminal stage, and two dashed curves represent the maturation of normal NK cells (left) and developmental process of malignant NK cells (right). Comparisons were made using Student's *t*‐test, and results for growth curves and bar plots are shown as mean value ± standard deviation (SD). ^ns^ *p* ≥ 0.05, *p < 0.05, **p < 0.01, ***p < 0.001, ****p < 0.0001. Scale bar, 100 µm.

We identified a total of 28 031 malignant NK cells grouped into six clusters with distinct expression signatures (**Figure** **2**A, Figure S6A and Table S3a, Supporting Information). To explore their potential cellular functions, we performed signaling pathway enrichment analyses using GSVA and Metascape. These analyses revealed specific enrichment of signaling pathways for each cell cluster, such as cell cycle and DNA replication signaling pathways related to cell proliferation in NK_C9_CXCL13 and NK_C10_STMN1, and cellular response to heat stress in NK_C7_CCL4 (Figure S6B,C, Supporting Information). These analyses also revealed upregulation of multiple cancer‐related pathways in malignant NK cells, including hypoxia, angiogenesis, CTLA4, NF‐κB, and inflammation signaling pathways, but downregulation of NK cell‐mediated cytotoxicity and its related leptin, nectin, protein secretion, and vesicular transport signaling pathways,^[^ ^30^, ^31^ ^]^ compared to normal NK cells (Figure S6B,C, Supporting Information). Furthermore, SCENIC analysis revealed distinct alterations in transcription factor (TF) activities between malignant and normal NK cells, including prominently upregulated activities of *MYC*, *NFKB1*, and *NFKB2* in malignant cell clusters (Figure S6D, Supporting Information), whose contribution to NKTCL tumorigenesis has been documented.^[^ ^15^ ^]^ Together, these observations highlight the heterogeneity of malignant NK cells with diverse biological functions and intrinsic malignant nature in NKTCL.

### 2.3 Pivotal Role of LMP1^+^ Malignant NK Cell Cluster in the Tumorigenesis and Development of NKTCL

Given the implication of EBV in NKTCL tumorigenesis,^[^ ^15^, ^22^ ^]^ we examined the expression of EBV‐encoded genes among malignant NK cell clusters (Figure S7A, Supporting Information). Interestingly, we observed that NK_C9_CXCL13 cluster uniquely expressed *LMP1*, a well‐known oncogene of EBV, in contrast to the other EBV genes (Figures S6A and S7B,C, Supporting Information). This finding was corroborated by the colocalization of LMP1 and CXCL13 in individual cells according to immunofluorescence (IF) staining assay from an independent NKTCL collection of Southern Chinese (SC‐cohort; Figure 2B and Figure S7D, Supporting Information) and a positive correlation between *LMP1* and *CXCL13* expression in the malignant NK cells in our scRNA‐seq cohort and tumor samples from another NKTCL collection in Shanghai^[^ ^15^ ^]^ (SH‐cohort; Figure S7E, Supporting Information).

We also observed a profoundly high expression of *STAT5A*, *STAT5B*, *NFKB1*, *NFKB2*, and *NFKBIA* in the NK_C9_CXCL13 cluster (Figure 2C). These genes are essential components of the JAK‐STAT and NF‐κB signaling pathways, which have been implicated in the oncogenesis of NKTCL^[^ ^32^ ^]^ and other EBV‐associated malignancies.^[^ ^21^, ^33^ ^]^ Consistently, SCENIC analysis revealed upregulated TF activities in the oncogenic JAK‐STAT (*STAT3* and *STAT5A*) and NF‐κB (*NKFB1*, *NKFB2*, and *RELB*) pathways in NK_C9_CXCL13 (Figure S6D, Supporting Information). Notably, the activation of these pathways was significantly correlated with *LMP1* expression (Figure S7F, Supporting Information). Furthermore, qPCR and western blotting assays revealed higher expression of *STAT3* and *STAT5B* in the LMP1^+^ NKTCL cell line (YT) compared to LMP1^−^ ones (KHYG‐1 and NK‐92; Figure S8A,B, Supporting Information). Moreover, the knockdown or overexpression of LMP1 led to the downregulation or upregulation of the JAK‐STAT and NF‐κB pathways, respectively (Figure S8C–F, Supporting Information). We also noted that LMP1 knockdown significantly inhibited cell proliferation and induced apoptosis in NKTCL cells (YT; Figure S9A–C, Supporting Information). Remarkably, with mouse models (Figure 2D), we observed a significant reduction of tumorigenic capacity, inhibition of cell proliferation, and an increase in apoptosis upon stable knockdown of LMP1 by lentivirus (Figure 2E–G) or adeno‐associated virus (AAV; Figure S9D–G, Supporting Information) in NKTCL cells. These findings strongly suggest that LMP1 is a critical oncogenic factor for NKTCL and that the NK_C9_CXCL13 subset, conferred by LMP1, may contribute to the tumorigenicity for NKTCL.

To further explore the potential development of NKTCL, we performed pseudotime trajectory analysis and identified a developmental branch from normal naïve NK cells (NK_C1_SELL) to the LMP1^+^ malignant NK cells (NK_C9_CXCL13; Figure 2H). This developmental path was concurrent with the expression of EBV‐encoded genes and alterations in transcriptional profiles in malignant cells (Figure S10A,B, Supporting Information). These observations suggest a potential origin of LMP1^+^ malignant cells from naïve NK cells in NKTCL, consistent with the similarity between normal NK cells at an earlier stage and NKTCL cells.^[^ ^34^ ^]^ Furthermore, we observed a developmental path from NK_C9_CXCL13 to other malignant NK cells (Figure 2H), along with the downregulation of *LMP1* (Figure S10C, Supporting Information). Consistently, RNA velocity analysis revealed a similar developmental trend across the heterogeneous malignant NK cells, with specific genes exhibiting transcriptional dynamics at each stage (Figure S10D,E, Supporting Information), suggesting their potential contributions to the transition of malignant NK cells. These observations jointly suggest a potential origin and developmental path of heterogeneous malignant NK cells, whereby LMP1^+^ NK_C9_CXCL13 cells play a pivotal role in the tumorigenesis and malignant progression of NKTCL.

### 2.4 Malignant NK Cells Regulate the Recruitment of Immune Cells Forming TME

**Figure 3:**
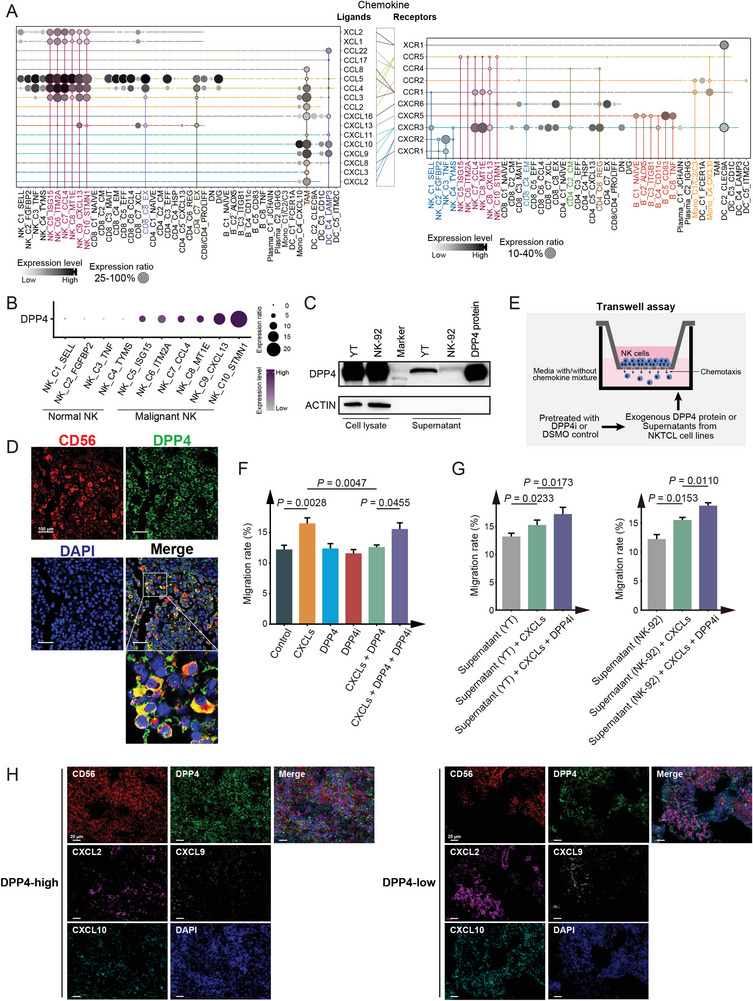
Chemotaxis regulation by malignant NK cells in NKTCL TME. A) Dot plots showing the expression of chemokine ligands (left panel) and receptors (right panel) among malignant NK cells and immune cells (indicated at the bottom) in NKTCL. Horizontal lines of identical color connect cells expressing ligands with cells expressing corresponding cognate receptors, and vertical lines highlight the expression patterns of chemokines in selected cell clusters. Cell clusters and chemokines are indicated at the *x*‐ and *y*‐axis, respectively. B) Dot plot showing the expression of *DPP4* among malignant and normal NK cell clusters (*x*‐axis). Circle sizes represent the proportions of cells expressing *DPP4*, and filled colors from light gray to deep purple represent normalized expression levels from low to high. C) Western blotting assay showing the protein expression of DPP4 and ACTIN in the supernatants and cell lysates of NKTCL cell lines (YT and NK‐92) with external DPP4 protein as positive control. D) Multiplex IF staining for DPP4‐expressing malignant NK cells (CD56^+^DPP4^+^) in NKTCL biopsies from the SC‐cohort. CD56 and DPP4 proteins as well as nuclear DNA are detected with different colors as indicated on top. Images are representative of biological replicates from three patients. Scale bars, 100 µm. E) Experimental design for the transwell assay to determine the migration ability of NK cells. DPP4 protein and the supernatants from NKTCL cells (YT and NK‐92) were incubated with DPP4 inhibitor (DPP4i) or DMSO as a control for 1 h, which were then incubated with culture medium without chemokines (PBS) or containing chemokine mixture for a 6‐h pretreatment and subsequently added into lower chambers. Peripheral NK cells isolated from PBMCs were then placed into the upper chambers for migration test. Bar plots showing the effect of F) exogenous or G) endogenous DPP4 on the transwell migration rates of NK cells. For (F), NK cells were cultured with chemokine mixture (CXCLs), and/or DPP4, and/or the DPP4i or without these factors (Control) in the lower chambers. For (G), NK cells were cultured with the supernatants from NKTCL cell lines (YT and NK‐92) with or without CXCLs and DPP4i in the lower chambers. Migration rates represent the percentages of migrated cells in all NK cells. Comparisons were made using paired Student's t‐test, and results are shown as mean value ± SD. H) Multiplex IF staining for DPP4 protein in malignant NK cells (CD56^+^) and extracellular regions as well as related chemokines (CXCL2, CXCL9, and CXCL10) in NKTCL tissue samples. The samples were categorized into two groups based on DPP4 expression: DPP4‐high (left panel) and DPP4‐low (right panel). Images are representative of biological replicates from three patients. Scale bars, 20 µm.

To explore potential mechanisms underlying the infiltration of immune cells forming NKTCL TME, we analyzed the transcriptional profiles of chemokines and their corresponding intercellular interactions between cell clusters (**Figure** **3**A, Table S4, Supporting Information). We observed that malignant NK cells exhibited extremely high expression of chemokines, such as *CCL3*, *CCL4*, and *CCL5*, which interacted with their corresponding receptors *CCR1* and *CCR5* widely expressed by various immune cells (Figure 3A, Figure S11A, Supporting Information). This suggests that these chemokines contribute to the chemoattractiveness of malignant cells in recruiting immune cells into NKTCL TME. Additionally, TAM had a remarkable expression of specific chemokines interacting with receptors *CCR1* and *CCR2* on peripheral monocytes (Mono_C1/C2/C3; via *CCL2*/*CCL3*/*CCL8*), *CXCR2* and *CXCR3* on normal NK cells (via *CXCL2*/*CXCL9*/*CXCL10*), and *CXCR3* on T cells (via *CXCL9*/*CXCL10*; Figure 3A), suggesting that TAMs may recruit both monocytes and cytotoxic immune cells from peripheral blood.

Notably, multiple chemokines, including *CXCL2*, *CXCL9*, and *CXCL10*, were predicted to interact with *DPP4* from malignant NK cells (Figure S11A, Supporting Information), which is a dipeptidylpeptidase capable of cleaving and degrading these chemokines in cancers.^[^ ^35^, ^36^ ^]^ Consistently, we observed a widespread expression of *DPP4* in malignant NK clusters (Figure 3B), which was confirmed by both secreted and cellular protein expression through western blotting in NKTCL cells and IF staining assays in NKTCL tumors from our SC‐cohort, respectively (Figure 3C,D). Transwell assays (Figure 3E) revealed that a mixture of *CXCL2*, *CXCL9*, and *CXCL10* induced the chemotaxis of freshly isolated normal NK cells, which was attenuated by DPP4 (Figure 3F). Furthermore, the addition of DPP4 inhibitor (DPP4i; Linagliptin) rescued the chemoattract activity of the chemokine mixture treated with DPP4 (Figure 3F) and significantly promoted the migration of NK cells into lower chambers with medium containing the chemokine mixture and NKTCL supernatant (Figure 3G). Supportively, further multiplex IF staining assays of the SC‐cohort revealed limited protein expression of CXCL2, CXCL9, and CXCL10 in patients with high expression of DPP4 (DPP4‐high), in contrast to their substantial expression in patients with low expression of DPP4 (DPP4‐low; Figure 3H). These observations strongly suggest that malignant NK cells may impair the recruitment of peripheral normal NK cells into NKTCL TME through secreting DPP4 to degrade CXCLs.

### 2.5 Malignant NK Cells and TAMs Jointly Foster an Immunosuppressive TME

**Figure 4:**
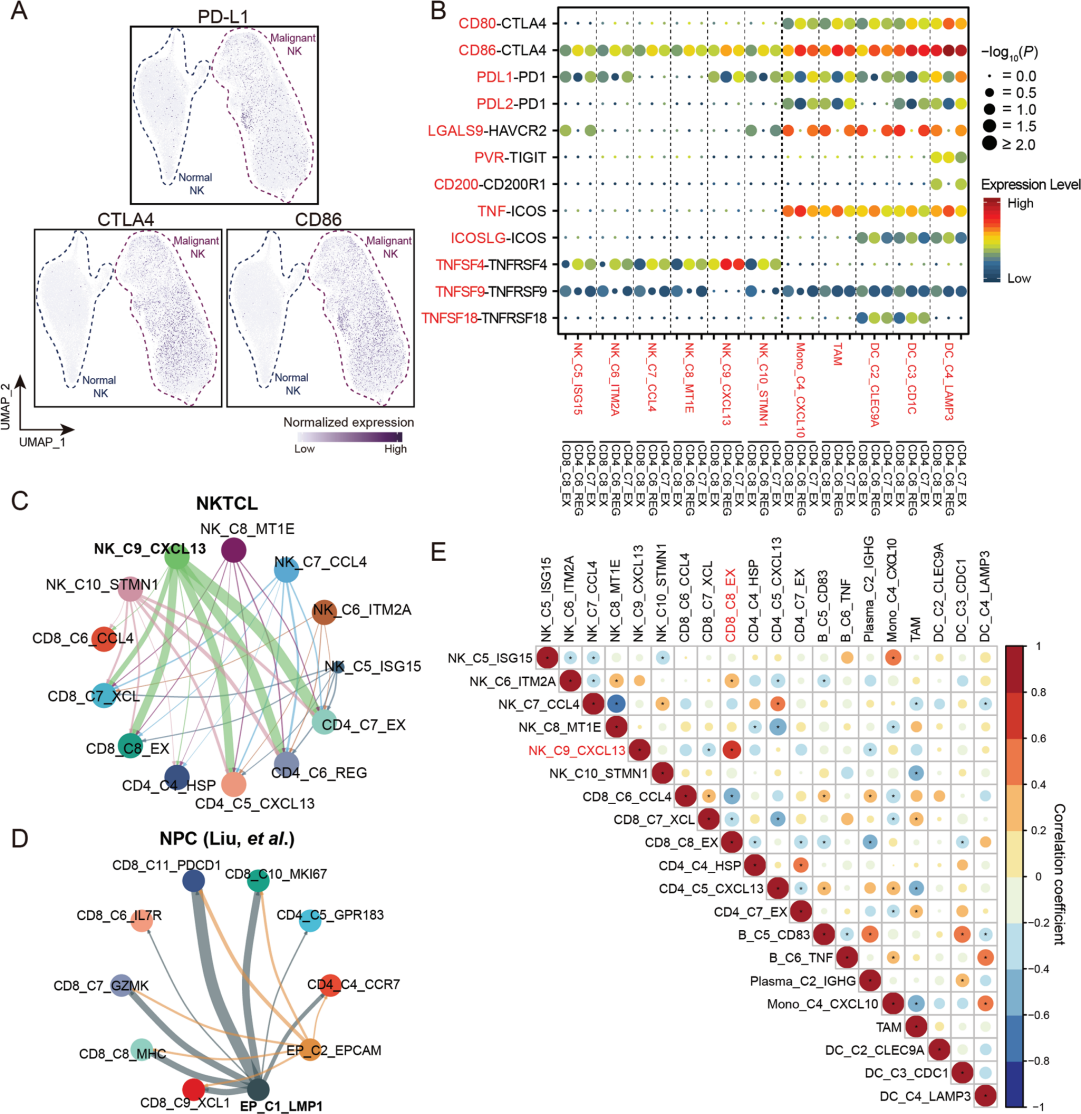
The immunosuppressive networks fostered by malignant NK cells in NKTCL TME. A) UMAP plots related to Figure 2A showing the normalized expression of immune checkpoint molecules, PD‐L1, CTLA4, and CD86, among normal and malignant NK cells. Each dot represents a cell, and the depth of color from light gray to deep purple represents low to high expression. B) Dot plot showing the ligand–receptor interactions (rows) of co‐stimulatory and co‐inhibitory molecules with significant difference between selected cell clusters (columns; the major left and right divisions annotated by the vertical dashed line in bold for malignant NK and myeloid cells, respectively). Ligands (red at the left; row) expressed by source cells (red at the bottom; column) are identified to interact with receptors (black at the left; row) expressed by exhausted and regulatory T cells (CD8_C8_EX, CD4_C6_REG, and CD4_C7_EX; black at the bottom; column). *P* values estimated using one‐sided permutation test (−log10 scaled) are indicated by circle sizes and the means of the average expression levels of two interacting molecules are indicated by filled colors, with blue to red representing low to high expression. Cellular interaction networks of immunosuppressive interactions between malignant NK cell clusters and tumor‐infiltrating T cell clusters in C) NKTCL and D) NPC. The thickness of each line represents the interaction intensity in scale estimated between the corresponding two cell types. E) Correlations between the cell fractions of pair‐wise cell clusters for NKTCL samples from the SH‐cohort (*n* = 51). The levels of correlation coefficients are indicated by filled colors; estimated *p* values are indicated by circle sizes; significant correlations are labeled (*p < 0.05).

Given that cancer cells can suppress T cells through expressing multiple immune checkpoint molecules,^[^ ^37^ ^]^ we examined the expression of these molecules in malignant cell clusters compared to normal NK cells. We observed a broad upregulation of *CTLA4* in malignant cell clusters, and functional enrichment analysis revealed the involvement of “CTLA4 signaling pathway” (**Figure** **4**A, Figure S6B, Supporting Information). Notably, we observed a predominant expression of *PD‐L1* and *CD86* in LMP1^+^ NK_C9_CXCL13 cells, consistent with their highest enrichment of “PD‐1 signaling pathway” (Figure 2C; Figure S6B, Supporting Information), suggesting their prominent immunosuppressive property. We also observed a significant correlation of expression between *LMP1* and *PD‐L1* or *CD86* (Figure S11B, Supporting Information). To further validate these findings, we performed qPCR and western blotting assays in NKTCL cell lines. The LMP1^+^ NKTCL cell line (YT) exhibited higher expression of both PD‐L1 and CD86 compared to the LMP1^−^ ones (KHYG‐1 and NK‐92; Figure S8A,B, Supporting Information). Moreover, knockdown or overexpression of LMP1 resulted in a decrease or increase in *PD‐L1* and *CD86* expression, respectively (Figure S8C–F, Supporting Information), which is consistent with a previous study.^[^ ^38^ ^]^ These observations strongly suggest an immunosuppressive potential of malignant NK in NKTCL, which LMP1 further enhanced.

**Figure 5:**
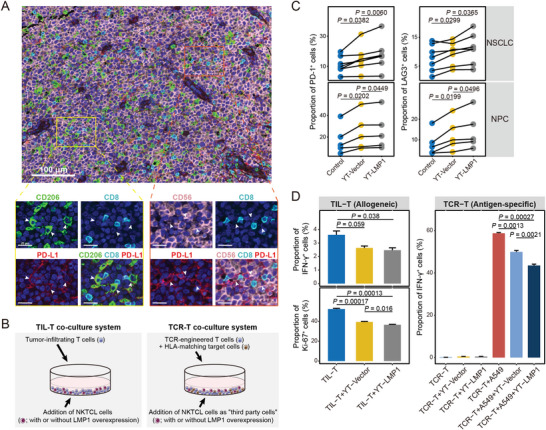
The immunosuppressive effect of malignant NK cells on T cells. A) Multiplex IF staining for the juxtaposition of PDL1‐expressing TAMs (CD206^+^PD‐L1^+^; indicated by white arrows in bottom left panels) or PDL1‐expressing malignant NK cells (CD56^+^PD‐L1^+^; indicated by white arrows in bottom right panels) with CD8^+^ T cells (CD8^+^) in NKTCL tissue samples from the SC‐cohort. Proteins of interest are indicated on top with different colors. Images are representative of biological replicates from three patients. Scale bars, 100 µm for the top panel and 20 µm for the bottom panels, respectively. B) Experimental design for the co‐culture assay to assess the immunosuppressive effect of malignant NK cells (with or without LMP1 overexpression) on T cells in allogeneic (TIL‐T system; left panel) and antigen‐specific ways (TCR‐T system; right panel). For the TIL‐T co‐culture system, tumor‐infiltrating T lymphocytes (TIL‐T) isolated from tumor tissues were co‐cultured with NKTCL cells (YT) with or without overexpression of LMP1 for two days. For the TCR‐T co‐culture system, T cells engineered with NY‐ESO‐1‐specific T cell receptor (TCR) were co‐cultured with their target cells (HLA‐matching A549/A2‐NY‐ESO‐1 cells), and additional YT cells were added as the “third‐party cells.” C) Line charts showing the proportions of PD1^+^ (left panel) and LAG3^+^ cells (right panel) in T cells upon different co‐culture treatments within T‐TIL system. Fresh tumor‐infiltrating T cells from non‐small‐cell lung cancer (NSCLC) or NPC were co‐cultured without (Control) or with NKTCL cells (YT) infected with lentivirus‐expressing *LMP1* (YT‐LMP1) or empty vector control (YT‐Vector). D) Bar plots showing the proportions of IFN‐γ^+^/Ki‐67^+^ cells in tumor‐infiltrating T cells (TIL‐T system; left panel) and TCR‐engineered T cells (TCR‐T system; right panel) upon different co‐culture treatments. Fresh tumor‐infiltrating T cells derived from tumor tissues (TIL‐T) or NY‐ESO‐1‐specific TCR‐engineered T cells (TCR‐T) with their HLA‐matching target cells (A549) were co‐cultured without or with NKTCL cells (YT) infected with lentivirus‐expressing LMP1 (YT‐LMP1) or empty vector control (YT‐Vector). Comparisons were made using Student's *t*‐test, and results for bar plots are shown as mean value ± SD. Corresponding treatments and the proportions of cells for the results of co‐culture assays are indicated at the *x*‐ and *y*‐axis, respectively.

Among the intercellular interactions between malignant cells and other TME cells, NK_C9_CXCL13 cells had the most remarkable interactions with other cells (Figure S11C,D and Table S4, Supporting Information). Notably, the LMP1^+^ malignant cluster showed the highest immunosuppressive interacting activity with exhausted and regulatory T cells through the interactions of CD86‐CTLA4 and PDL1‐PD1 (Figure 4B,C). This observation is consistent with our finding in nasopharyngeal carcinoma (NPC; Figure 4D), further indicating the most substantial immunosuppressive potential of NK_C9_CXCL13 cells among the malignant NK clusters. Supportively, we observed the strongest positive correlation between the fractions of LMP1^+^ NK_C9_CXCL13 cells and exhausted CD8^+^ T cells (Figure 4E). Furthermore, multiplex IF staining assay revealed physical juxtapositions of PDL1‐expressing malignant NK cells (CD56^+^PD‐L1^+^) and CD8^+^ T cells in NKTCL tumors from our SC‐cohort (**Figure** **5**A).

To validate the effect of LMP1^+^ malignant NK cells on T cell suppression, we first performed co‐culture assays using an allogeneic approach, involving NKTCL cells and tumor‐infiltrating T cells (TIL‐T system; Figure 5B). We observed upregulated expression of PD‐1 and LAG3 but suppressed expression of IFN‐γ and Ki‐67 in T cells upon co‐culture with NKTCL cells, and these changes were more significant when T cells were co‐cultured with LMP1‐overexpressed NKTCL cells (Figure 5C,D). Furthermore, we also conducted co‐culture assay using an antigen‐specific approach, a TCR‐T system consisting of T cells engineered with NY‐ESO‐1‐specific TCR and HLA‐matching A549/A2‐NY‐ESO‐1 tumor cells, where NKTCL cells were added to evaluate their immunosuppressive effect on the T cells (Figure 5B). Notably, the addition of NKTCL cells significantly decreased IFN‐γ expression within the T cells, which was further suppressed by LMP1‐overexpressed NKTCL cells (Figure 5D). These observations suggest a more robust immunosuppressive capacity of LMP1^+^ malignant NK cells on T cells than their LMP1^−^ counterparts.

Among the broad intercellular interactions between immune cells, we observed the most intensive interactions between TAMs and other cell types (Figure S11C,D and Table S4, Supporting Information). Notably, TAMs had putative interactions with tumor‐infiltrating T cells through CD80/CD86‐CTLA4, PDL1/PDL2‐PD1, and LGALS9‐HAVCR2 (Figure 4B), suggesting their immunosuppressive capability on T cells. Supportively, multiplex IF staining assays also revealed physical juxtapositions of PDL1‐expressing TAMs (CD206^+^PD‐L1^+^) and CD8^+^ T cells (CD8^+^) in NKTCL tumors from our SC‐cohort (Figure 5A). Taken together, these observations revealed a robust immunosuppressive intercellular network in NKTCL TME, where malignant NK cells (especially LMP1^+^ ones) and TAMs jointly mediate intense immunosuppression on T cells, thereby perturbing the immune landscape of NKTCL.

### 2.6 Heterogeneous TME Components as Prognostic Indicators for NKTCL Patients

**Figure 6:**
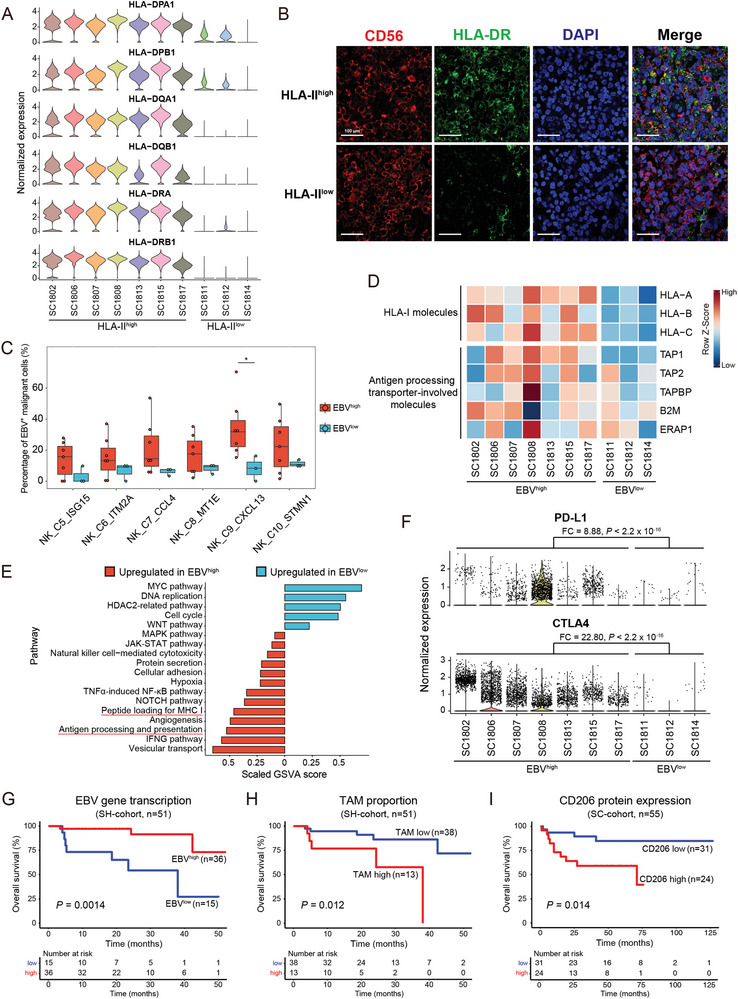
Heterogeneous TME signatures and the prognostic indicators of NKTCL. A) Violin plots showing the normalized expression of HLA‐II genes in malignant NK cells for each patient. Patients of two groups and the expression levels of genes are indicated at the *x*‐ and *y*‐axis, respectively. B) Multiplex IF staining for HLA‐II‐expressing malignant NK cells (CD56^+^HLA‐DR^+^) in NKTCL tumor biopsies of patients with HLA‐II^high^ (top panel) or HLA‐II^low^ (bottom panel) from the SC‐cohort. CD56 and HLA‐DR proteins as well as nuclear DNA are detected with different colors as indicated on top. Images are representative of biological replicates from three patients. Scale bar, 100 µm. C) Box plot showing the proportion of EBV^+^ cells (*y*‐axis) for each malignant NK cluster (*x*‐axis) in patient groups of EBV^high^ (red) or EBV^low^ (blue). Center lines denote median values; whiskers denote 1.5× the interquartile range; colored dots denote the proportion of EBV^+^ cells for each malignant cluster in each patient. Comparison was made using Wilcoxon rank‐sum test. D) Heatmap showing the expression levels of selected genes (rows) in malignant NK cells for each patient from our scRNA‐seq cohort (columns). Filled colors from blue to red in the squares represent normalized expression levels from low to high as scaled in row direction (row Z‐score). E) Bar plot showing signaling pathways with significant difference between EBV^high^ and EBV^low^ patients. Each pathway is colored according to its upregulation in either EBV^high^ (red) or EBV^low^ (blue) samples. The scaled GSVA scores and pathways are indicated at the *x*‐ and *y*‐axis, respectively. F) Violin plots showing the normalized expression levels (*y*‐axis) of PD‐L1 (top panel) and CTLA4 (bottom panel) in malignant NK cells for each patient (*x*‐axis). Comparison between two groups was made using Wilcoxon rank‐sum test. Kaplan–Meier overall survival curves of NKTCL patients stratified according to their transcriptional levels of G) EBV‐encoded genes or fractions of H) TAMs in the SH‐cohort (*n* = 51) or protein expression levels of CD206 in the SC‐cohort (*n* = 55; I). The red and blue lines represent two groups of patients with high and low levels of indicated TME signatures. Survival duration and probability are indicated at the *x*‐ and *y*‐axis, respectively. *P* values were calculated using log‐rank test. The bottom tables show the numbers of patients at risk by time for two groups.

We observed varied proportions of each cell cluster across patients (Figure 1E; Table S2, Supporting Information), indicating individual heterogeneity in NKTCL as other cancer types (Figure S4A, Supporting Information). Considering the involvement of HLA class II (HLA‐II) genes in NKTCL,^[^ ^15^, ^18^, ^19^ ^]^ we examined their cellular expression and revealed two distinct groups of patients with high (HLA‐II^high^; *n* = 7) and low (HLA‐II^low^; *n* = 3) transcription of HLA‐II genes in malignant NK cells (**Figure** **6**A), which was further corroborated by IF staining assay (Figure 6B). Consistently, we observed a high degree of CNVs at the MHC region encompassing HLA‐II genes in malignant NK cells from both groups (Figure S12A, Supporting Information) and a significant upregulation of *CIITA*, the master regulator of HLA‐II genes,^[^ ^39^ ^]^ in malignant cells from the HLA‐II^high^ patients (Figure S12B, Supporting Information). Furthermore, we observed differential transcription of EBV‐encoded genes in the malignant NK cells from the HLA‐II^high^ and HLA‐II^low^ groups, with higher transcription in the former (EBV^high^) and lower transcription in the latter (EBV^low^) (Figure S12C,D, Supporting Information), suggesting inter‐patient heterogeneity of EBV gene transcription in NKTCL. Moreover, we observed a higher proportion of EBV‐infected cells in each malignant NK cell cluster from the EBV^high^ samples compared to the EBV^low^ samples, with a significant increase in the NK_C9_CXCL13 cluster (Figure 6C). Supportively, we also identified EBV^high^ and EBV^low^ samples in the independent validation SH‐cohort (*n* = 51; Figure S12E, Supporting Information).

Given that EBV could induce the expression of HLA class I (HLA‐I) genes, thereby enhancing the immunogenicity of cancer cells through endogenous antigen processing pathway,^[^ ^40^ ^]^ we further evaluated the expression of HLA‐I and other related molecules. Notably, we observed upregulation of all these genes in malignant NK cells from the EBV^high^ samples (Figure 6D), which is consistent with their functional enrichment in “antigen processing and presentation” and “peptide loading for MHC I” pathways (Figure 6E). Furthermore, we observed a positive correlation between EBV gene transcription and immunogenic potential in the malignant cells in both our scRNA‐seq cohort and the SH‐cohort (Figure S12F, Supporting Information). These observations together suggest a link between enhanced immunogenicity and activated EBV infection of malignant cells in EBV^high^ NKTCL patients.

Cellular interaction analysis revealed more abundant cellular interactions between malignant cells and immune cells in the EBV^high^ samples (Figure S13A and Table S5, Supporting Information), including significantly upregulated interactions of PDL1‐PD1, CD86‐CTLA4, and CD80‐CTLA4 (Figure S13B–D, Supporting Information). Consistently, we observed significantly higher expression of *PD‐L1* and *CTLA4* in malignant NK cells and stronger exhaustion of tumor‐infiltrating T cells in the EBV^high^ samples (Figure 6F; Figure S14A, Supporting Information). These observations suggest a higher immunosuppressive potential of malignant cells from EBV^high^ patients. Supportively, we noted a significant correlation between increased exhaustion score and high transcription of EBV‐encoded genes in the SH‐cohort (Figure S14B, Supporting Information). Furthermore, the EBV^high^ samples in the SH‐cohort exhibited higher proportions of exhausted CD8^+^ T cells (fold change = 1.72; CD8_C8_EX), indicating a stronger immunosuppressive TME (Figure S14C, Supporting Information).

Survival analysis of the SH‐cohort revealed that NKTCL patients with EBV^high^ had significantly better overall survival (OS; *n* = 51; *p* = 0.0014; Figure 6G), indicating that high transcription of EBV‐encoded genes in tumor is a favorable prognostic marker. Additionally, a higher fraction of TAMs was associated with worse OS (*p* = 0.012; Figure 6H) and higher expression of TAM marker CD206 protein was also associated with worse OS in the SC‐cohort (*n* = 55; *p* = 0.014; Figure 6I; Figure S15A, Supporting Information). These observations suggest high infiltration of TAMs as an unfavorable prognostic marker in NKTCL.

In addition, we further explored the associations between clinical characteristics (such as age, gender, and tumor stage) and TME components of NKTCL in our scRNA‐seq patient cohort. Interestingly, we observed a significant reduction of regulatory (CD4_C6_REG) T cells, which have immunoregulatory properties implicated in multiple cancers,^[^ ^37^ ^]^ in patients with advanced stages of NKTCL (Figure S15B–D, Supporting Information), suggesting their potential contribution to NKTCL progression.

## 3 Discussion

**Figure 7:**
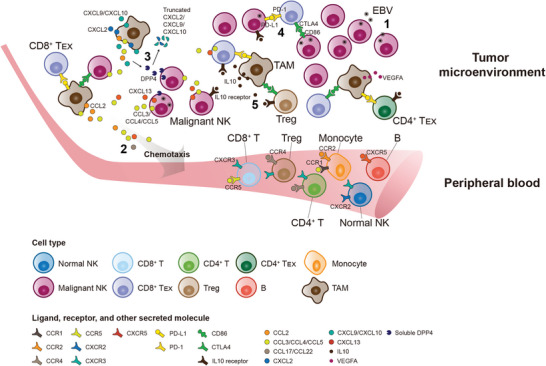
Schematic diagrams of cross‐talks among malignant NK cells and immune cells in the TME of NKTCL. EBV‐infected malignant NK cells and immune cells together participate in the development of NKTCL. **1**) Upon EBV infection, LMP1 may contribute to the malignant transformation of NK cells (Figure 2H). **2**) Malignant NK cells and TAMs secret a variety of chemokines (including CCL2, CCL3, CCL4, CCL5, etc.) and thereby recruit multiple types of immune cells from peripheral blood through corresponding chemotactic interactions (Figure 3A). **3**) Soluble DPP4 secreted by malignant NK cells can truncate and rapidly degrade CXCL2, CXCL9, and CXCL10 in NKTCL TME, whereby hampering the recruitment of CXCR2^+^CXCR3^+^ immune cells (Figure 3B–H). **4**) Malignant NK cells (especially LMP1^+^ ones) expressing CD86 and PD‐L1 can negatively regulate the immune response of tumor‐infiltrating T cells including exhausted and regulatory T cells (CD8^+^ T_EX_, CD4^+^ T_EX_, and Treg; Figures 4A–C and 5A–D). **5**) TAMs not only secrete immunosuppressive IL10 and angiogenic VEGFA, but also interact with tumor‐infiltrating T cells through suppressive interactions of CD86‐CTLA4 and PDL1‐PD1 (Figures 4B and 5A).

For the first time to our knowledge, we conducted a comprehensive analysis to unravel the mechanisms by which malignant NK cells with EBV‐encoded LMP1 contribute to reshaping the cellular landscape and fostering an immunosuppressive niche of NKTCL TME at single‐cell resolution (**Figure** **7**). While EBV infection is a key pathological feature of NKTCL,^[^ ^1^ ^]^ its role in NKTCL tumor progression remains enigmatic. Previous studies have demonstrated that the heterogeneity of NKTCL can be attributed to various factors related to EBV, such as specific mutations, different EBV strains, and abnormal expression of viral genes,^[^ ^15^, ^22^ ^]^ partially explaining the diverse treatment outcomes observed in patients with NKTCL. In our study, we identified six distinct malignant NK cell clusters characterized by differential expression of EBV‐encoded genes. These clusters exhibited unique transcriptomic signatures, enriched pathways, and distinct transcriptional regulation, which align with the heterogeneous hallmarks of cancer and potentially correlate with their developmental stages along the pseudotime trajectory. Considering the crucial roles of different EBV genes in tumor progression coping with the EBV life cycle,^[^ ^20^, ^41^ ^]^ our findings suggest the heterogeneity observed in malignant NK cell clusters may be influenced by the varied expression of EBV genes, ultimately promoting NKTCL tumorigenesis and its malignant progression.

Among the various viral genes encoded by EBV, LMP1 is a well‐known tumorigenic factor that has been implicated in tumor progression, invasion, and metastasis in multiple types of cancers.^[^ ^20^ ^]^ In our study, we identified a specific malignant NK cluster (NK_C9_CXCL13) marked with the unique expression of *LMP1*, which was presented at the central stage in the developmental process from normal NK to heterogeneous malignant cells. Furthermore, the LMP1^+^ malignant NK cells showed upregulation of the JAK‐STAT and NF‐κB signaling pathways, both of which have known oncogenic functions in NKTCL^[^ ^15^, ^32^ ^]^ and other cancers.^[^ ^21^, ^33^ ^]^ Notably, these pathways were further boosted upon LMP1 overexpression. These observations suggest that LMP1 may be a key driver inciting tumorigenesis and the subsequent malignant progression of NKTCL through oncogenic activation and other transcriptional dysregulation. This is consistent with its early tumorigenic role reported in the malignant transformation of B cells and epithelial cells in other EBV‐associated malignancies.^[^ ^20^, ^42^ ^]^ Supportively, previous studies in NKTCL have also linked LMP1 to the survival, proliferation, and even chemoresistance of cancer cells through the activation of MAPK and NF‐κB pathways.^[^ ^38^, ^43^ ^]^

Although LMP1 has been previously associated with immune evasion by downregulating the major histocompatibility complex and inducing the expression of PD‐L1,^[^ ^21^ ^]^ the specific immunosuppressive function of LMP1^+^ malignant cells on T cells in EBV‐associated cancers remains poorly understood. In our study, we observed distinct expression of immune checkpoint molecules, *CD86* and *PD‐L1*, in the LMP1^+^ NK_C9_CXCL13 cells, which further exhibited the most intensive immunosuppressive interactions of CD86‐CTLA4 and PDL1‐PD1 with T cells. Furthermore, our study demonstrated a positive correlation between LMP1 expression and the expression of CD86 and PD‐L1, potentially due to the oncogenic activation of NF‐κB pathway.^[^ ^21^, ^38^, ^44^ ^]^ Noteworthily, our study also demonstrated that malignant NK cells, particularly those expressing LMP1, have immunosuppressive functions. These findings suggest that these malignant cells exhibit immunosuppression likely through upregulating these immune checkpoint molecules and inducing corresponding interactions with T cells, thereby perturbing the immune landscape of NKTCL TME. Therefore, targeting LMP1^+^ malignant cells could be a potential therapeutic strategy for NKTCL. Indeed, our in vitro and in vivo experiments showed that LMP1 knockdown through shRNA and AAV vectors effectively suppressed cell proliferation and tumor growth in NKTCL cells. These findings are consistent with the promising efficacy of RNA interference‐based therapies in other EBV‐associated cancers. ^[^ ^45^, ^46^, ^47^ ^]^ In addition, T‐cell therapy targeting LMP1 has shown promising efficacy in EBV‐associated lymphomas,^[^ ^48^, ^49^ ^]^ including a recent in vivo CAR‐T investigation of NKTCL.^[^ ^50^ ^]^

Intensive infiltration of immune cells is a common characteristics of NKTCL,^[^ ^28^ ^]^ thereby establishing the heterogeneous cellular architecture of TME.^[^ ^51^, ^52^ ^]^ Notably, cell–cell interaction analysis revealed intensive interactions between malignant NK cells and immune cells through *DPP4* and multiple chemokines. Furthermore, our data showed that DPP4 secreted from malignant NK cells could attenuate the chemotaxis of CXCR2^+^CXCR3^+^ normal NK cells, potentially explaining the lower infiltration of normal NK cells in NKTCL compared to other cancers.^[^ ^29^, ^53^, ^54^, ^55^ ^]^ Given that *DPP4* participates in diminishing the recruitment of T cells and eosinophils from peripheral blood through cleaving their interacting chemokines,^[^ ^35^, ^56^ ^]^ its broad expression by malignant NK cells may be an intriguing mechanism for restraining the infiltration of not only normal NK cells but also other immune cells, orchestrating TME with impaired anti‐tumor immunity in NKTCL. Noteworthily, considering the significant increase of chemotaxis of NK cells upon the addition of DPP4 inhibitor, blockade of DPP4 may enhance the tumoral infiltration of normal NK cells and other immune cells in NKTCL, providing a novel therapeutic strategy for NKTCL.

Our findings unveil that malignant NK cells tangle with TAMs and T cells to foster an immunosuppressive niche within NKTCL TME, a robust strategy allowing cancer to avoid immune destruction.^[^ ^24^ ^]^ We note that malignant NK cells have upregulated transcription of the PD‐1 pathway, enabling them to regulate exhausted T cells through immunosuppressive interaction of PDL1‐PD1. These observations may explain promising outcomes upon PD‐1 blockade treatment^[^ ^10^ ^]^ and suggest that this therapeutic strategy should be considered earlier for patients with NKTCL. Interestingly, we also observed a broader expression of *CTLA4* in malignant NK cells and tumor‐infiltrating T cells. Furthermore, the interactions of CD80/CD86‐CTLA4 between malignant NK cells or TAMs and T cells were more pronounced compared to the PDL1‐PD1 axis. Therefore, we suspect that CTLA4 blockade could be a viable salvage therapeutic approach for patients with NKTCL less benefited from anti‐PD1 blockade therapy. Alternatively, combination therapy with both CTLA4 and PD1 blockade, similar to the strategy used in melanoma,^[^ ^57^ ^]^ may be effective. However, these hypotheses await further clinical investigations.

Profiling the heterogenous TME signatures provides valuable insights into patient stratification for precise treatment.^[^ ^58^, ^59^ ^]^ Our study revealed two groups of NKTCL patients with high and low expression of EBV‐encoded genes. These groups are equivalent to high and low HLA‐II expression in malignant NK cells, which is consistent with their known involvement in NKTCL development.^[^ ^15^, ^18^, ^19^ ^]^ EBV^high^ tumors with high HLA‐II expression in malignant NK cells may have a profound immune activation, since NK cells with upregulation of HLA‐II genes could enhance the immune response of CD4^+^ T cells through antigen presentation.^[^ ^60^ ^]^ Furthermore, EBV^high^ tumors had higher immunogenicity potential, as reflected by higher transcription of HLA‐I molecule and enrichment of endogenous antigen processing pathway. These data suggest that the high transcription of EBV‐encoded genes in malignant NK cells induces antigen presentation processing, provoking an immune response by cytotoxic cells to strengthen anti‐tumor immunity^[^ ^40^, ^61^, ^62^ ^]^ and consequently leading to better survival outcomes for NKTCL patients. Intriguingly, EBV^high^ tumors had a stronger immunosuppressive TME, which might be a consequence of the persistent exposure of tumor‐infiltrating T cells to high level of EBV antigens upon long‐term viral infection.^[^ ^37^ ^]^ Therefore, we suspect that immune checkpoint blockade therapies may be particularly beneficial for EBV^high^ patients with intensive immunosuppression and yet retaining high immunogenicity in their tumors.

In summary, through comprehensively uncovering the heterogeneous TME of NKTCL at single‐cell resolution, we identified specific malignant and immune cell clusters, essential molecules, and complex interacting networks for NKTCL, which provide insights into understanding the pathogenic mechanisms of NKTCL and developing novel immunotherapeutic approaches, as well as identifying prognostic biomarkers for patient stratification in this deadly disease. We acknowledged that our study has several limitations. First, our single‐cell analysis leaves out the NKTCL with a T‐cell lineage, a minor group of NKTCL,^[^ ^1^ ^]^ thus differing from the three molecular subtypes identified by a previous study based on bulk somatic mutations and transcriptional signatures of patients with NKTCL.^[^ ^15^ ^]^ Second, although we obtained sufficient cells and sequencing reads for each sample in our single‐cell analysis, increasing the sample size would allow for a better exploration of cell subsets with minor proportions and their roles in NKTCL. Third, it would be more informative to validate our findings in larger cohorts, including diverse ethnic and regional groups. Lastly, further investigation using single‐cell and spatial transcriptome analyses is awaited to explore the changes in the heterogeneous TME components of NKTCL under different treatment regimens, thereby help identify critical events and inform the development of better therapeutic strategies.

## 4 Experimental Section

### Patients and Study Design

For scRNA‐seq analysis, 10 patients with NKTCL were enrolled at Sun Yat‐sen University Cancer Center (SYSUCC), Guangzhou, China (Table S1, Supporting Information). Fresh tumor biopsy samples and matching peripheral blood samples were collected for each patient, followed immediately by single cell preparation as described below. An independent NKTCL sample cohort was also collected from Shanghai (SH‐cohort) with bulk transcriptome data and corresponding prognostic information. ^[^ ^15^ ^]^ For IF and immunohistochemistry (IHC) assays, formalin‐fixed paraffin‐embedded (FFPE) specimens from another independent NKTCL collection of southern Chinese (SC‐cohort) were collected from SYSUCC and National Cancer Centre of Singapore (NCCS), Singapore (Table S6, Supporting Information).

All patients were newly diagnosed and histopathologically confirmed according to the WHO classification criteria for NKTCL (the cytotoxic, CD3ε‐positive, and EBER‐positive phenotype) and did not have a history of cancer or a concurrent malignancy at the time of recruitment. This study was approved by the institutional review boards (B2019‐223‐01) and conducted under the guidance of the Declaration of Helsinki. Written informed consent was obtained from all participants.

### Sample Processing and Single Cell Collection

Each fresh tumor sample was treated with enzymatic digestion and mechanical dissociation to generate single cell suspension immediately after receiving tumor samples, according to the previous procedures with minor modifications.^[^ ^29^, ^63^ ^]^ Briefly, fresh tumor samples were cut into ≈1‐mm^3^ pieces and enzymatically digested with Collagenase II (Gibco, USA; Cat #17101015) and IV (Gibco; Cat #17104019) for 30–40 min on a rotator at 37 °C. Subsequently, the digested mixtures were passed through a 40 µm Cell‐Strainer (BD Biosciences, USA; Cat #352340) in the Dulbecco's Modified Eagle Medium (DMEM; Gibco) with 10% fetal bovine serum (FBS; Gibco) and uniform cell suspensions were obtained. The suspended cells were then centrifuged at 400×*g* for 5 min and resuspended using 0.8% NH_4_Cl red blood cell lysis buffer (RBCL; Sigma‐Aldrich, USA; Cat #254134‐5G) with further incubation on ice for 10 min to lyse red blood cells. After washing twice with DPBS (Gibco; Cat #14190250), the pelleted cells were resuspended in sorting buffer, consisting of DPBS supplemented with 0.04% BSA (Sigma‐Aldrich; Cat #9048468).

For blood samples, 5 mL of fresh peripheral blood was transferred into EDTA anticoagulant tubes (BD Biosciences; Cat #366643) and subsequently layered onto HISTOPAQUE‐1077 solution (Sigma‐Aldrich; Cat #10771). After centrifugation, PBMCs remained at the plasma–solution interface were carefully collected and wash twice with DPBS. Red blood cells were removed via the same procedure abovementioned.

Viable cells from both tumor and blood samples were identified with negative staining of propidium iodide (Invitrogen, USA; Cat #P1304MP) and collected using fluorescence activated cell sorting (FACS; BD FACSAria III; BD Biosciences).

### Single‐Cell Library Construction and Sequencing

Viable cells were subjected for droplet‐based single‐cell cDNA library construction using Chromium Single Cell 5′ Library Construction Kit (10× Genomics, USA) according to the manufacturer's instructions. Briefly, targeting a capture of 8000 cells per sample, appropriate volume of cell suspension with a concentration of 700–1200 cells µL was loaded in each channel, which was further mixed with barcoded gel beads on a Chromium Controller (10× Genomics). mRNA transcripts were reverse‐transcribed into cDNA with barcoded indexes at 5′‐end, followed by cDNA amplification on a thermal cycler (C1000; Bio‐Rad, USA). Amplified cDNA was then used for 5′ gene expression library construction. Lastly, libraries were sequenced with pair‐end reads of 150 bp using Illumina HiSeq X Ten instruments (Illumina, USA).

### Single‐Cell RNA Sequencing Data Processing

Droplet‐based sequencing data were aligned and quantified using Cell Ranger (version 3.0.2) against the human reference genome (GRCh38) and EBV reference sequence (Akata),^[^ ^64^ ^]^ with force‐cells parameter of 13 000 and other parameters by default unless otherwise stated. On average, more than 360 million sequencing reads and more than 80% of sequencing saturation were obtained for each sample, and 1345 genes and 3813 unique molecular identifiers (UMIs) on average were obtained for each cell, indicating sufficient coverage and representation of transcriptome.

For each sample, raw gene expression matrix in the “filtered_feature_bc_matrix” file folder generated from Cell Ranger count was converted to a Seurat object using the Seurat package (version 3.2.3)^[^ ^65^ ^]^ in R (version 3.6.2). For quality control, low quality cells (UMI ≤ 1000, gene number ≤ 500, and mitochondrial genome fragments ≥ 0.15) and genes with extremely rare frequency (expressed in less than 10 cells) were removed. R package DoubletFinder (version 2.0.2)^[^ ^66^ ^]^ was applied to detect and remove doublets for each sample, with an expected doublet rate of 7.5% and other parameters by default (Figure S1B, Supporting Information). The remaining cells were normalized at logarithm using NormalizeData function and scaled using ScaleData function with regressing out heterogeneity associated with the amount of UMIs and percentage of mitochondrial genes. Subsequently, gene expression matrices for all samples were merged, and batch effects between patients were removed using FindIntegrationAnchors and IntegrateData function in Seurat. After dimensional reduction using RunUMAP function with top 30 dimensions, cells were clustered into subsets using FindClusters function with a resolution of 1.0 and default parameters used otherwise. An overlapping distribution of cell clusters across patients was observed, suggesting that the potential batch effect due to the transcriptional variance across individuals was minimal in the analyses (Figure S1C, Supporting Information). Canonical marker genes were used for the annotation of major cell types, while several other minor cell types (endothelial cells, epithelial cells, fibroblasts, smooth muscle cells, etc.; Figure S1D, Supporting Information) were excluded from further analyses due to their minimal quantities.

To further identify cell subtypes, the abovementioned procedure (data normalization and scaling, dimensional reduction, and cell clustering) was repeated for each major cell type (NK, T, B, and myeloid cells) with same parameters except for setting top 20 dimensions in dimensional reduction. Differential expression genes with significant positive expression on average (logFC > 0.25, *p* < 0.05) in each cluster were identified using FindAllMarkers function, which were subsequently used as markers to annotate cell clusters.

### CNV Inference

To identify malignant cells among NK cell clusters, potential somatic alterations of large‐scale chromosomal CNVs, either gains or losses, were estimated using R package inferCNV (version 1.2.1).^[^ ^67^ ^]^ Using transcription data of normal NK cells from the previous single‐cell study as reference,^[^ ^29^ ^]^ the CNV landscape of 10 NK cell clusters was generated with default parameters. For a better inference, cell clusters with more than 500 cells were downsampled to 500. The CNV levels of all T cell clusters were also estimated in the same manner.

Besides the high degree of CNVs, malignant NK cells were also identified with their unique expression of EBV‐encoded genes (*LMP1*, *LMP2*, *RPMS1*, *EBER1*, and *EBER2*) and high expression of histologically diagnostic markers (*PRF1* and *GZMB*).

### Collection of Public Single‐Cell RNA Sequencing Datasets

To compare the transcriptional signatures and cell compositions of NKTCL tumor with other cancers and healthy tissues, additional scRNA‐seq datasets that are publicly available were collected, including datasets for non‐small cell lung cancer (NSCLC),^[^ ^54^ ^]^ head and neck squamous cell carcinoma,^[^ ^55^ ^]^ cutaneous T‐cell lymphoma,^[^ ^68^ ^]^ and human cell landscape,^[^ ^69^ ^]^ as well as the in‐house NPC^[^ ^29^ ^]^ and adult human cell atlas^[^ ^63^ ^]^ datasets.

### Calculation of Functional Module Scores

To evaluate the potential capacities of specific functions across different cell clusters, functional module scores were calculated for cell clusters using AddModuleScore function in Seurat at single‐cell level. Each module was calculated with its corresponding gene set (Table S7, Supporting Information). EBV gene transcriptional scores were calculated to estimate the expression levels of EBV‐encoded genes in malignant NK cells from HLA‐II^high^ and HLA‐II^low^ samples, considering specific EBV infection of malignant NK cells.

### Functional Enrichment Analysis

For signaling pathway enrichment analysis, gene set variation analysis (GSVA) was applied to identify enriched or depleted gene sets across cell clusters, using R package GSVA (version 1.34.0)^[^ ^70^ ^]^ with default parameters. Metascape^[^ ^71^ ^]^ was also used, by selecting top 100 differential expressed genes for each cell cluster with logFC > 0.25 and p.adjust < 0.05, to determine significantly enriched “gene ontology” and “Reactome” terms.

### Transcriptional Regulator Analysis

To infer gene regulatory networks, TFs with different activities across NK cell clusters were identified using R package SCENIC (version 1.1.2.2).^[^ ^72^ ^]^ Using Wilcoxon rank‐sum test, top 10 significantly upregulated TFs were displayed for each malignant or normal NK cell cluster.

### Developmental Trajectory Analysis

To construct a potential developmental process among cell clusters, R package Monocle3 (version 0.2.1)^[^ ^73^ ^]^ was performed for NK and myeloid cells. For a better inference, cell clusters with more than 500 cells were downsampled to 500. Raw gene expression matrices were imported, following the general pipeline implemented in Monocle3. For a better performance of dimensional reduction and spatial configuration, umap.min_dist parameter of 0.8 and umap.n_neighbors parameter of 5 L were set for reduce_dimension function. Subsequently, cells were ordered in trajectory projection, of which pseudotime scores were calculated with naïve cell cluster specified as root node. When analyzing across NK cell clusters, NK_C3_TNF and NK_C5_ISG15 cells were eliminated in consideration of their small quantities (*n* = 89 and 144, respectively) and distinctive enrichment in one patient (NK_C3_TNF in SC1812).

### RNA Velocity Analysis

To trace the cell fate of malignant NK cells, RNA velocity and transcriptional dynamics were estimated through R package velocyto.R (version 0.6). ^[^ ^74^ ^]^ The result of UMAP dimensional reduction from the abovementioned Monocle3 was used for cell embedding. Directional flows generated by velocyto.R were used to infer the developmental destination for nearby malignant NK cells.

### Cellular Communication Analysis

CellPhoneDB software (version 2.0.6)^[^ ^75^ ^]^ was applied to identify the ligand‐receptor pairs across malignant and immune cell clusters in both tumor and peripheral blood. With high similarity of upregulated interaction pairs, monocytes from blood (Mono_C1_VCAN, Mono_C2_TXNIP, and Mono_C3_FCGR3A) were assigned as Mono_C1/C2/C3 throughout cellular communication analysis. For a better inference, cell clusters with more than 500 cells were downsampled to 500. Ligands and receptors detected in more than 5% cells in the tested cell clusters were kept (–threshold 0.05). Moreover, the statistical significance of each interaction pair was estimated through 1000 permutations (–iterations 1000).

### Bulk RNA Sequencing Data Analysis and Survival Analysis

Transcriptional data using RNA sequencing of bulk tissue and clinical data of 51 patients with NKTCL of NK cell origin were retrieved from a previous study (the SH‐cohort).^[^ ^15^ ^]^ The expression profiles were normalized as transcripts per million to exclude potential bias. Normalized expression levels of five EBV‐encoded genes involved in the abovementioned module score calculation (*LMP1*, *LMP2*, *RPMS1*, *BNRF1*, and *EBER2*; Table S7, Supporting Information) were used to divide 51 patients into EBV^high^ and EBV^low^ samples in the SH‐cohort through k‐means clustering. Subsequently, Kaplan–Meier curve was conducted to estimate the difference of survival probability between EBV^high^ and EBV^low^ patients.

The fractions of 20 cell clusters mainly observed in NKTCL tumor rather than peripheral blood, such as NK_C9_CXCL13, CD8_C8_EX, and TAM, were estimated by CIBERSORTx (https://cibersortx.stanford.edu)^[^ ^76^ ^]^ for each bulk sample. Correlations between cell fractions were estimated through Spearman's rank correlation test and comparison between EBV^high^ and EBV^low^ samples was made for the fraction of each cell cluster through Wilcoxon rank‐sum test. To reveal the association between the fraction of TAMs and survival of patients, a receiver operating characteristic curve was used to determine an optimal cut‐off value, by which patient cohorts were grouped into two groups with high and low fractions of TAMs. Then, Kaplan–Meier estimator was conducted to reveal the prognostic ability of TAMs in the SH‐cohort, and a log‐rank test was performed to compare the survival outcomes between two groups.

### Cell Culture and Reagents

The human embryonic kidney 293T cells were purchased from Cell Bank of Type Culture Collection of Chinese Academy of Sciences, Shanghai Institute of Cell Biology, Chinese Academy of Sciences, which were cultured in DMEM supplemented with 10% FBS. All human NKTCL cell lines (YT, KHYG‐1, and NK‐92) were originated from NCCS and maintained at SYSUCC. YT cells were cultured in Iscove's Modified Dulbecco's Medium (Gibco) supplemented with 20% FBS, and 1% Sodium pyruvate; KHYG‐1 cells were cultured in RPMI 1640 Medium (Gibco) supplemented with 10% FBS; NK‐92 cells were cultured in RPMI 1640 Medium supplemented with 10% FBS, 10% Horse Serum (Gibco), and recombinant human IL‐2 (Miltenyi Biotec, Germany; Cat #130‐097‐746). Genetically modified human lung adenocarcinoma A549 cells expressing NY‐ESO‐1 protein in the context of HLA‐A*0201 (A549/A2‐NY‐ESO‐1) were kindly gifted by Professor Penghui Zhou at SYSUCC, which were cultured in RPMI1640 medium supplemented with 10% FBS. All cell lines were maintained under standard cell culture conditions at 37 °C in a water‐saturated atmosphere of 5% CO_2_. No evidence of mycoplasma contamination was observed using mycoplasma detection kit (Vazyme, China; Cat #D101‐02). All primary antibodies are commercially available, of which information is provided in Table S8 (Supporting Information).

### Quantitative PCR Assay

Total RNA was extracted from NKTCL cell lines using Trizol reagent (Invitrogen), and reverse transcription (RT) was performed using oligo (dT) primers and M‐MLV Reverse Transcriptase (Promega, USA; Cat #M1701) according to the manufacturer's instructions. Real‐time quantitative RT PCR (qRT‐PCR) was performed to determine the transcription level of genes using Premix Ex Taq Kit (Takata, Japan; Cat #RR390W) and their corresponding primer pairs (Table S9, Supporting Information). mRNA level of each gene was examined in three technical replicates.

### Stable Knockdown and Overexpression of Genes of Interest in NKTCL Cell Lines

Lentiviral expression system was deployed to manipulate the expression of *LMP1* in cells. For knockdown, DNA fragments of shRNAs targeting *LMP1* (primer sequences listed in Table S9, Supporting Information) were cloned into lentiviral vector pLKO.1 following the protocol by Addgene (https://www.addgene.org). For overexpression, full length of EBV *LMP1* cDNA was obtained using RT‐PCR and mRNA derived from Akata cells as template (Table S9, Supporting Information), and subsequently cloned into lentiviral vector pCDH. Lentiviral constructs were packaged and transfected into 293T cells to generate lentivirus. NKTCL cells were infected with the lentivirus and selected against puromycin.

### Western Blotting Assay

Western blotting assay was performed to determine protein expression using conventional method. In brief, protein lysates were prepared in cell lysis buffer (CST, USA) with protease inhibitor cocktail (Beyotime, China; Cat #P1005), followed by separation on sodium dodecyl sulfate‐polyacrylamide gel electrophoresis and transfer to polyvinylidene difluoride membrane (Merck Millipore, USA) with wet tank blotting (Bio‐Rad). After incubations with primary and secondary antibodies, signals were detected by using ChemiDoc Touch Imaging System (Bio‐Rad). Primary and secondary antibodies involved are listed in Table S8 (Supporting Information).

### Co‐Culture Assays

Co‐culture assays were also performed to assess the immunosuppressive effect of malignant NK cells (with or without LMP1 overexpression) on T cells in both allogenic and antigen‐specific approaches. For the former, due to the limited sample size of NKTCL tumor biopsy, tumor‐infiltrating T lymphocytes (TIL‐T) were isolated from fresh tumor tissues from patients with NPC and NSCLC (with five and seven independent replicates, respectively), which contained enough T cells for analysis. T cells were stimulated with anti‐CD3 (eBioscience; Cat #16‐0038‐85) and anti‐CD28 (eBioscience; Cat #16‐0288‐85) for two days and then co‐cultured with NKTCL cells (YT) with or without overexpression of *LMP1* for two days (thus named as TIL‐T system). The latter was defined as a TCR‐T system, where NY‐ESO‐1‐specific T cell receptor (TCR) was engineered into T cells, followed by coculture with target cells (HLA‐matching A549/A2‐NY‐ESO‐1 cells) and “third‐party cells” (NKTCL cells). Briefly, primary T cells were isolated using FACS from the peripheral blood cells of healthy donors collected at the Guangzhou Blood Center, China, and subsequently stimulated with anti‐CD3 (eBioscience) and anti‐CD28 (eBioscience) for two days. Activated T cells were then transduced with lentivirus to express a high‐affinity TCR, for the specific recognition of HLA‐A*0201‐restricted peptide sequence in the human testis cancer antigen NY‐ESO‐1. Five days after activation, TCR‐engineered T cells were assessed for transduction efficiency by quantifying the percentage of T cells expressing Thy1 using FACS with anti‐Thy1 antibody (Biolegend; Cat #328110), and subsequently incubated in the presence of NKTCL cells (YT) with or without overexpression of LMP1 at a ratio of 3:1 for 24 h. Finally, these TCR‐engineered T cells were co‐cultured with their target A549/A2‐NY‐ESO‐1 cells at a ratio of 3:1 for 24 h. Using FACS, T cells from co‐culture assays were stained and estimated with anti‐PD‐1 APC (Biolegend; Cat #329908) and anti‐LAG3 Alexa Fluor 700 (eBioscience, USA; Cat #56‐2239‐42) for exhausted status, anti‐IFN‐γ APC (eBioscience; Cat #17‐7319‐82) for cytotoxic activity, and anti‐Ki‐67 Brilliant Violet 421 (Biolegend, Cat #350505) for cell proliferation.

### Transwell Assay

Transwell assays were performed to determine the migration ability of NK cells through 3‐µm transwell filters (Corning, USA; Cat #3415) from the upper chambers to the medium in the lower chambers, with treatments of interest. Briefly, peripheral NK cells (CD3^−^CD56^+^) were isolated from PBMCs using FACS. After overnight stimulation with IL‐2, NK cells (1 × 10^5^) were placed into the upper chambers for migration test. DPP4 protein (Biolegend; Cat #764102) and the supernatants from NKTCL cells (YT and NK‐92) were incubated with DPP4 inhibitor (Linagliptin; Selleck; Cat #S3031) or DMSO (Sigma‐Aldrich; Cat #D4540) as control for 1 h, which were then incubated with culture medium without chemokines (PBS) or containing 100 ng mL^−1^ of chemokine mixture with CXCL2 (R&D Systems, USA; Cat #276‐GB‐010), CXCL9 (R&D Systems; Cat #392‐MG‐010), and CXCL10 (R&D Systems; Cat #266‐IP‐010) for a 6‐h pretreatment and subsequently added into lower chambers. Migrated NK cells were collected for cell counting after incubation at 37 °C and 5% CO_2_ for 3 h. Transwell assays were performed in seven independent replicates.

### Xenograft Mouse Model

Male BALB/c nude of four‐week‐old were purchased from Beijing Vital River Laboratory Animal Technology (China), and randomly divided into groups as indicated and maintained under a specific pathogen‐free condition (*n* = 6 for each group). Stable YT cell lines (5 × 10^6^) with the knockdown of LMP1 mediated by either lentivirus‐ or AAV‐expressing shRNAs (sh‐LMP1‐1/‐2 or AAV‐sh‐LMP1) and the respective control vectors (sh‐Luci or AAV‐control) were mixed with Matrigel (Corning) and subcutaneously injected into the dorsal flank of nude mice.

Tumor size was measured every week with a caliper, and the tumor volume was determined with the formula: *L* × *W* ^2^ × 0.5, where *L* is the longest diameter and *W* is the shortest diameter. Four weeks after the inoculation of YT cells, nude mice were euthanized, and in vivo xenografts were dissected for further analysis. All animal experiments were performed under protocols approved by the Institutional Animal Care and Use Committee at SYSUCC (L102012021223F).

### Immunostaining Assay

The paraffin blocks were cut into 5‐µm sections and adhered on the glass slides, followed by dewaxing, rehydration, blockade of endogenous peroxidase activity, and antigen retrieval at high‐temperature. Subsequently, the FFPE sections were processed further for either multiplex IF or IHC staining assays.

Multiplex IF staining assays were conducted to determine the presence of specific malignant NK cell clusters (NK_C9_CXCL13 and NK_C10_STMN1), expression of certain proteins (DPP4, HLA‐DR, GZMB, and PRF1) in malignant NK cells, and cell proliferation and apoptosis. Briefly, the sections were permeabilized in PBS with 0.1% Triton X‐100 (Sigma‐Aldrich; Cat #T8787) and incubated overnight at 4 °C with primary antibodies. Subsequently, the sections were incubated with goat anti‐rabbit IgG (Invitrogen; Cat #A‐11008) and goat anti‐mouse IgG secondary antibodies (Invitrogen; Cat #A‐11004). Nuclei were counterstained with 4′‐6′‐diamidino‐2‐phenylindole (DAPI; Sigma‐Aldrich; Cat #D9542). Images were captured using a confocal laser‐scanning microscope (LSM880; Zeiss, Germany). Primary antibodies are listed in Table S8 (Supporting Information). Each IF staining assay was conducted in three independent replicates.

To determine the spatial contact of malignant NK cells, TAMs, and CD8^+^ T cells, as well as the association between DPP4 and CXCL (CXCL2, CXCL9, and CXCL10) proteins in NKTCL tumors, multiplex IHC staining assays were performed using the PANO Multiplex IHC Kit (Panovue, China; Cat #0004100100) according to the manufacturer's instructions. Briefly, the slides were incubated with blocking reagent at room temperature for 10 min, and then incubated with primary antibodies overnight at 4 °C. The slides were then incubated with the secondary antibody (HRP polymer, goat anti‐mouse/rabbit IgG) at room temperature for 10 min. Subsequently, fluorophore (tyramide signal amplification or TSA plus working solution) was applied to the sections, followed by heat‐treatment with microwave. The primary antibodies were applied sequentially, followed by incubation with the secondary antibody and TSA treatment. Nuclei were stained with DAPI after all the antigens had been labelled. Multispectral images for each stained slide were captured using the Mantra System (PerkinElmer, USA). Primary antibodies are listed in Table S8 (Supporting Information). Each multiplex IHC staining assay was conducted in three independent replicates.

The protein expression of CD206 was examined using IHC staining of FFPE specimens from the SC‐cohort. The sections were incubated with the primary antibody against CD206 (anti‐CD206; CST, Cat #24595) followed by the incubation with a secondary antibody (Zsbio; Cat #PV‐6001). Next, the sections were stained with 3,3′‐diaminobenzidine (DAB; Zsbio; Cat #ZLI‐9017) and counterstained with hematoxylin (Beyotime; Cat #C0107‐100 mL) for nuclei. For the stained sections from the SC‐cohort, IHC scores were determined by the percentages of stained cells, as the intensities of color reaction for CD206 were similar among stained cells in NKTCL tissue biopsies. All NKTCL patients were then divided into two groups with high and low IHC scores of CD206 immunostaining, for further survival analysis.

### Statistical Analysis

Statistical analyses were performed using R, with methods as described in the Figure legends. Survival was measured using the Kaplan‐Meier method. Spearman's rank correlation test was used to estimate correlations among expression levels of genes, scores of functional modules, and fractions of cell types. Statistical significance was determined by two‐sided Student's *t*‐test, Wilcoxon rank‐sum test, and log‐rank test. *P* < 0.05 was considered as statistical significance.

## Conflict of Interest

The authors declare no conflict of interests.

## Author Contributions

Y.‐Q.L. and C.‐L.L. contributed equally to this work. J.‐X.B. and R.‐J.P. conceived and supervised this study; R.‐J.P., H.‐Q.H., Y.G., J.X., W.‐L.Z., C.K.O., S.T.L., X.‐X.W., C.L.C., J.Y.S.C., Y.H., and Y.X. provided clinical samples and information; S.H., Y.L., and Y.‐Q.L. performed sample preparation, library construction, and data generation for scRNA‐seq; Y.‐Q.L., S.H., Y.L., J.Q.L., and G.‐W.L. performed bioinformatics analyses; C.‐L.L., J.‐X.J., Y.‐Q.L., W.‐X.Y., Q.C., D.C.H., I.N.H., Y.‐H.L., D.M., Y.‐T.Z., and P.‐P.W. performed functional validations; Y.‐Q.L. and J.‐X.B. interpreted the results and wrote the manuscript. All authors read and approved the final manuscript.

## Supporting information

Supporting Information

Supplemental Table 1

Supplemental Table 2

Supplemental Table 3

Supplemental Table 4

Supplemental Table 5

Supplemental Table 6

Supplemental Table 7

Supplemental Table 8

Supplemental Table 9

## Data Availability

Processed single‐cell RNA Sequencing data of this study can be obtained from Gene Expression Omnibus (GEO) with an accession number of GSE203663. The key data in this study have also been deposited in the Research Data Deposit (RDD; http://www.researchdata.org.cn/) with an accession number of RDDB2023192723. The raw FASTQ files in this study will be provided for scientific research upon request complying with the law due to human patient privacy concerns. Code used for all processing and analysis is available upon request.
