# Supplementary material for: Single‐Cell Analysis Reveals Malignant Cells Reshape the Cellular Landscape and Foster an Immunosuppressive Microenvironment of Extranodal NK/T‐Cell Lymphoma
Source: Adv Sci (Weinh). 2023 Nov 10;10(36):2303913. doi: 10.1002/advs.202303913 (PMC10754138; doi:10.1002/advs.202303913)
Supplement: Supplementary file 1 — Supporting Information [file ADVS-10-2303913-s004.pdf]

ADVANCED SCIENCE
Open Access

Supporting Information

for *Adv. Sci.*, DOI 10.1002/advs.202303913

Single-Cell Analysis Reveals Malignant Cells Reshape the Cellular Landscape and Foster an Immunosuppressive Microenvironment of Extranodal NK/T-Cell Lymphoma

Yi-Qi Li, Chun-Ling Luo, Jia-Xin Jiang, Shuai He, Yang Liu, Wen-Xin Yan, Yi Xia, Qian Cui, Ying Huang, Jing Quan Lim, Dachuan Huang, Izzah Nabilah Hussein, Yan Gao, Guo-Wang Lin, Yi-Hong Ling, Dong Ma, Yue-Tong Zhang, Jason Yongsheng Chan, Pan-Pan Wei, Xiao-Xiao Wang, Chee Leong Cheng, Jie Xiong, Wei-Li Zhao, Choon Kiat Ong, Soon Thye Lim, Hui-Qiang Huang, Rou-Jun Peng and Jin-Xin Bei**

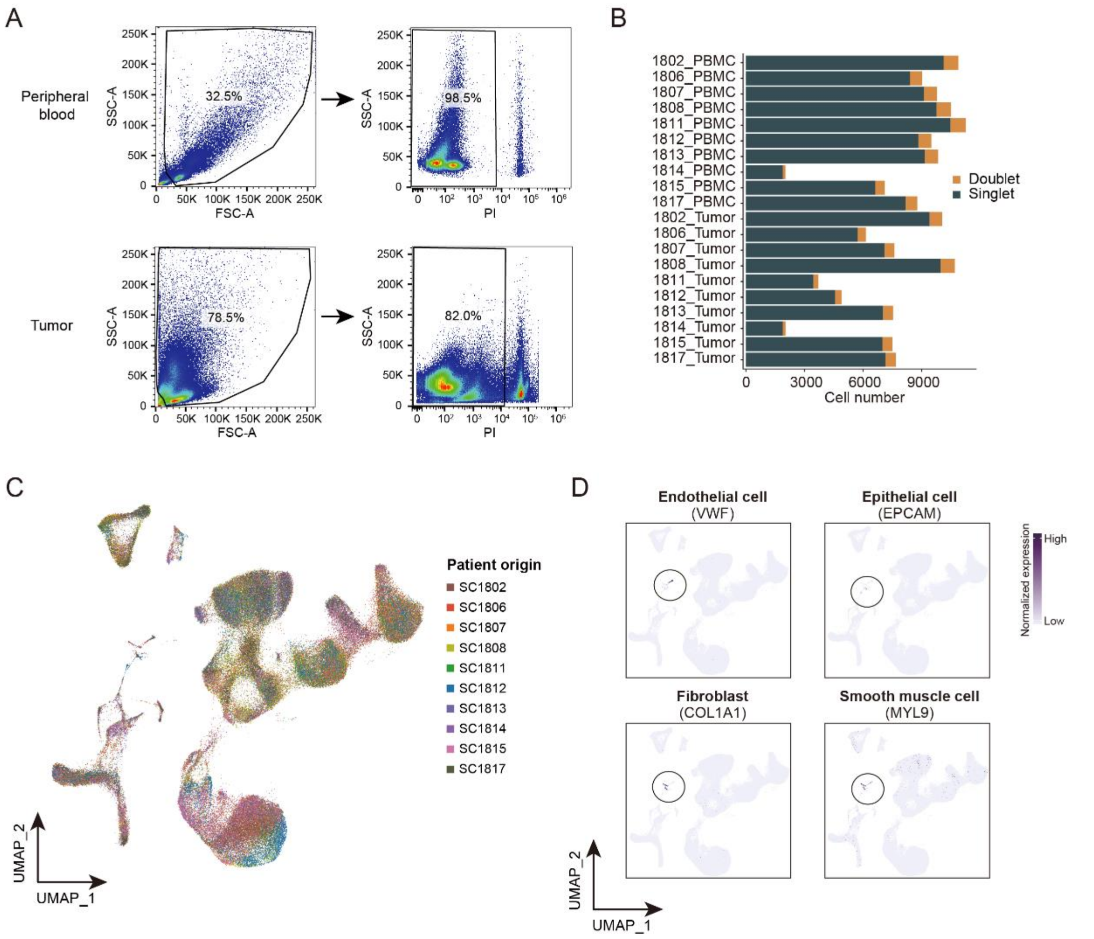

Figure S1 | Basic information of scRNA-seq data.

A. Representative images showing fluorescence-activated cell sorting (FACS) for cells (left panels) with viability (right panels) from peripheral blood (top panels) and tumor samples (bottom panels).

B. Bar plot showing the number (x-axis) of singlets (dark cyan) and doublets (orange) in each sample (y-axis). The naming system is as “[patient ID]_[tissue origin]”. For example, “1802_PBMC” and “1802_Tumor” represent PBMC and tumor tissue for the patient SC1802, respectively.

C. UMAP plot showing cells derived from ten patients. Each dot represents a cell, colored according to the patient it derived as indicated at the right panel. An overlapping distribution of cell clusters across patients represents minimal batch effect across individuals after batch removal.

D. UMAP plots showing the normalized expression of marker genes to define several types of non-immune cells with small quantities—endothelial cells (*VWF*), epithelial

cells (*EPCAM*), fibroblasts (*COL1A1*), and smooth muscle cells (*MYL9*). Each dot represents a cell, and the depth of color from light grey to deep purple represents low to high expression.

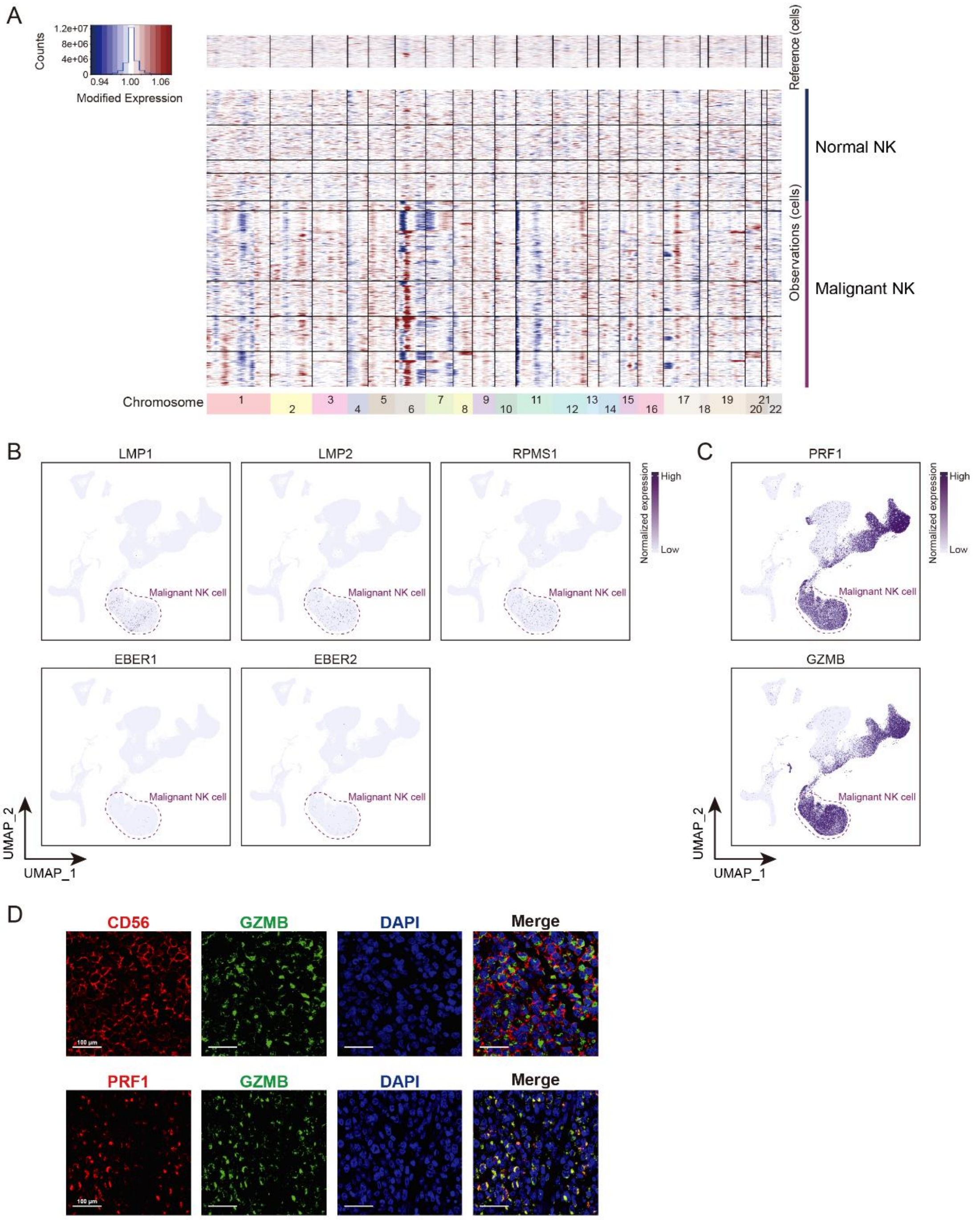

Figure S2 | Identification of malignant NK cells in NKTCL.

A. Heatmap showing the large-scale chromosomal CNVs in normal and malignant NK

cells, and reference cells (rows). CNVs are inferred as gains (red) or losses (blue) according to the average expression of 100 genes within each chromosomal region (columns).

B-C. UMAP plots showing the normalized expression of histologically diagnostic markers, including EBV encoded-genes (B) and cytotoxic molecules (C). Each dot represents a cell, and the depth of color from light grey to deep purple represents low to high expression.

D. Multiplex immunofluorescence staining for GZMB-expressing malignant NK cells ($CD56^{+}GZMB^{+}$; top panel) and co-expression of PRF1 and GZMB ($PRF1^{+}GZMB^{+}$; bottom panel) in NKTCL biopsies. The detection of CD56, GZMB, and PRF1 proteins as well as nuclear DNA are shown with different colors as indicated. Images are representative of biological replicates from three patients. Scale bars, 100 μm.

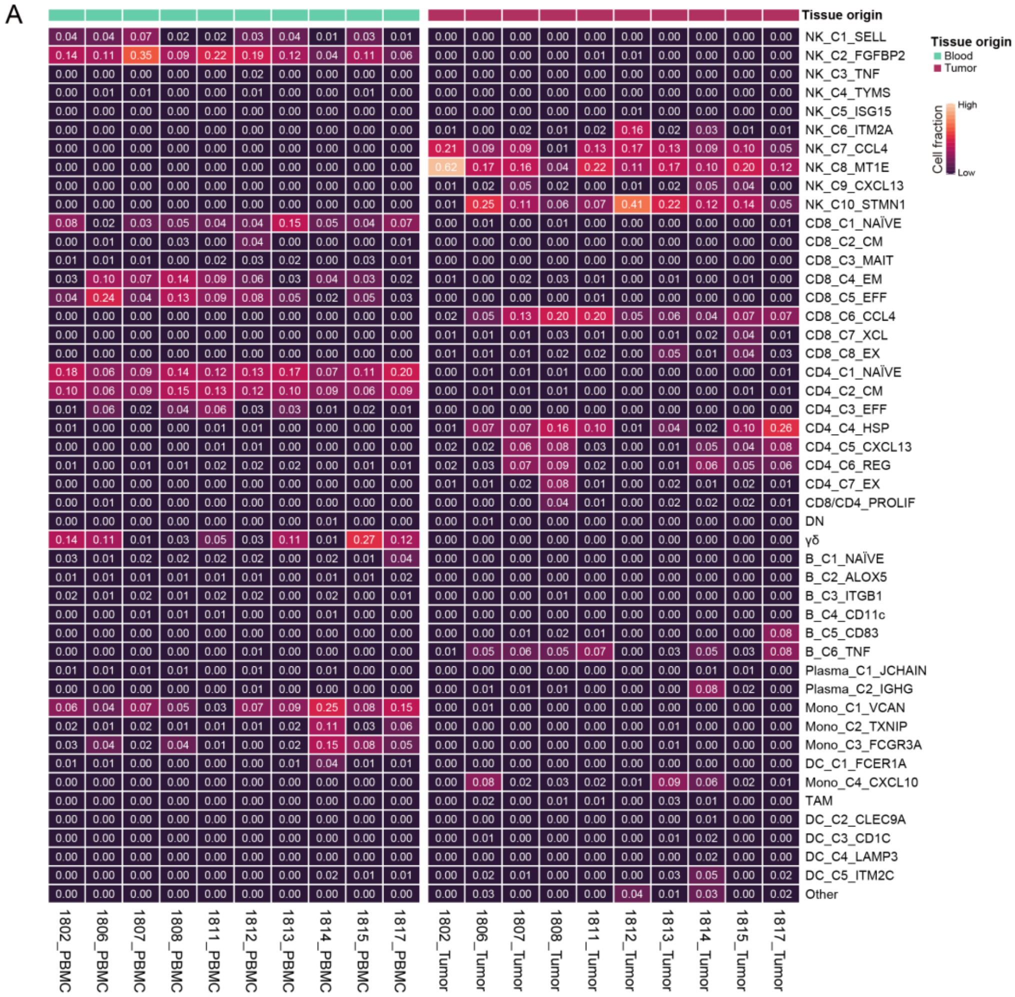

Figure S3 | Cell fractions for each blood and tumor samples of NKTCL.

A. Heatmap showing the cell fractions of each cell subcluster (rows) within each blood and tumor sample of NKTCL (columns), related to **Table S2**. Cell fractions for either blood (cyan) or tumor samples (dark red) are indicated as rectangles on top. Filled colors from deep purple to orange in the heatmap boxes represent cell fractions from low to high.

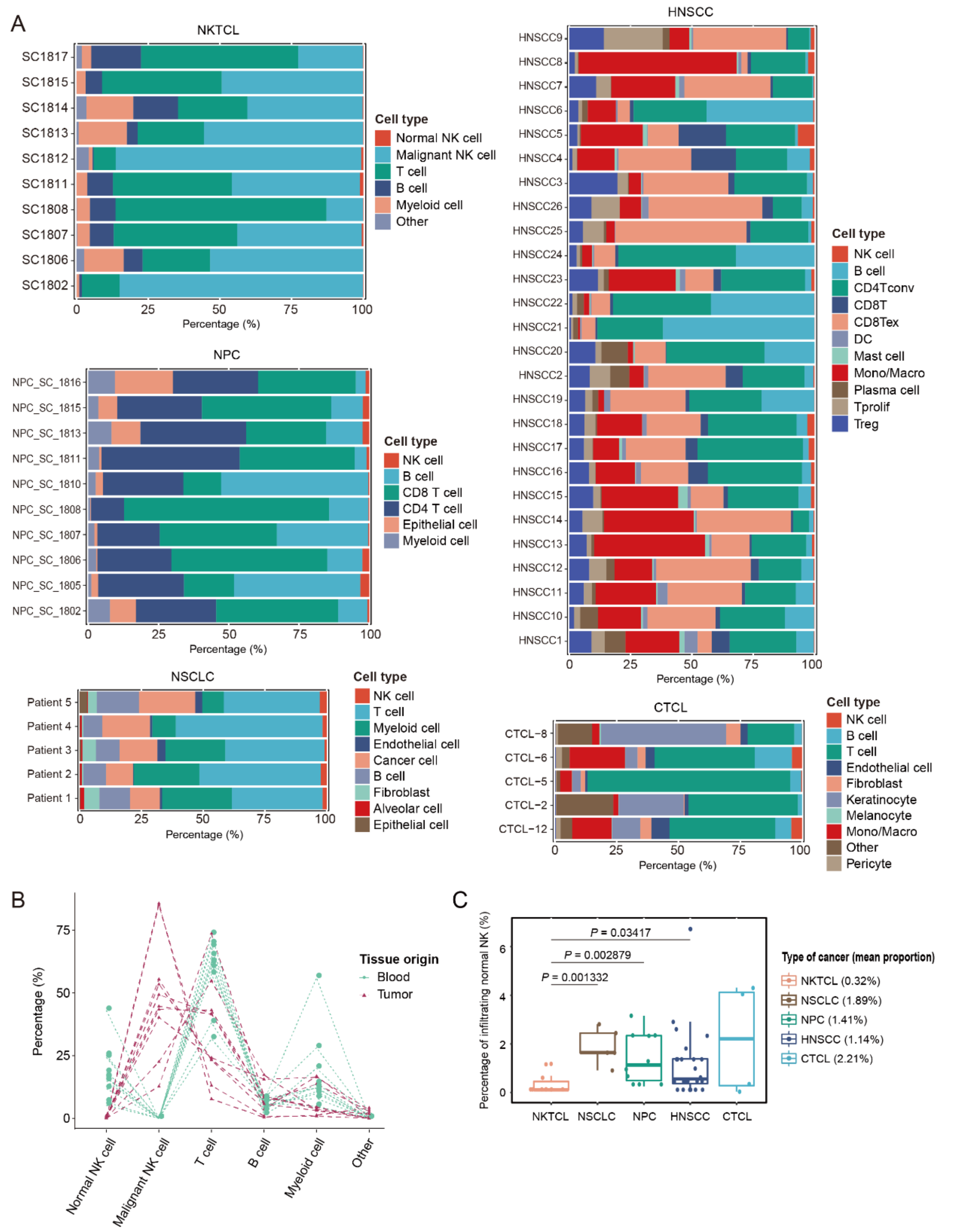

Figure S4 | Cellular composition of NKTCL and other types of cancers.

A. Bar plots showing the proportions of major cell types in NKTCL, NPC (nasopharyngeal carcinoma), NSCLC (non-small-cell lung cancer), HNSCC (head and neck squamous cell carcinoma), and CTCL (cutaneous T-cell lymphoma). Each bar represents an individual patient, with cell proportions colored according to cell types as indicated at the right panel.

B. Line chart showing the fractions (y-axis) of major cell types (x-axis) in each NKTCL patient, derived from either peripheral blood (cyan) or tumor tissue (dark red).

C. Box plot showing the proportion of infiltrating normal NK cells (y-axis) for each tumor sample of NKTCL, NSCLC, NPC, HNSCC, and CTCL (x-axis), with corresponding colors for cancer types at the right panel. Center lines denote median values, which are provided at the right panel; whiskers denote 1.5× the interquartile range; colored dots denote the proportion of infiltrating normal NK cells for each sample in different cancers. Comparisons were made using Wilcoxon rank-sum test.

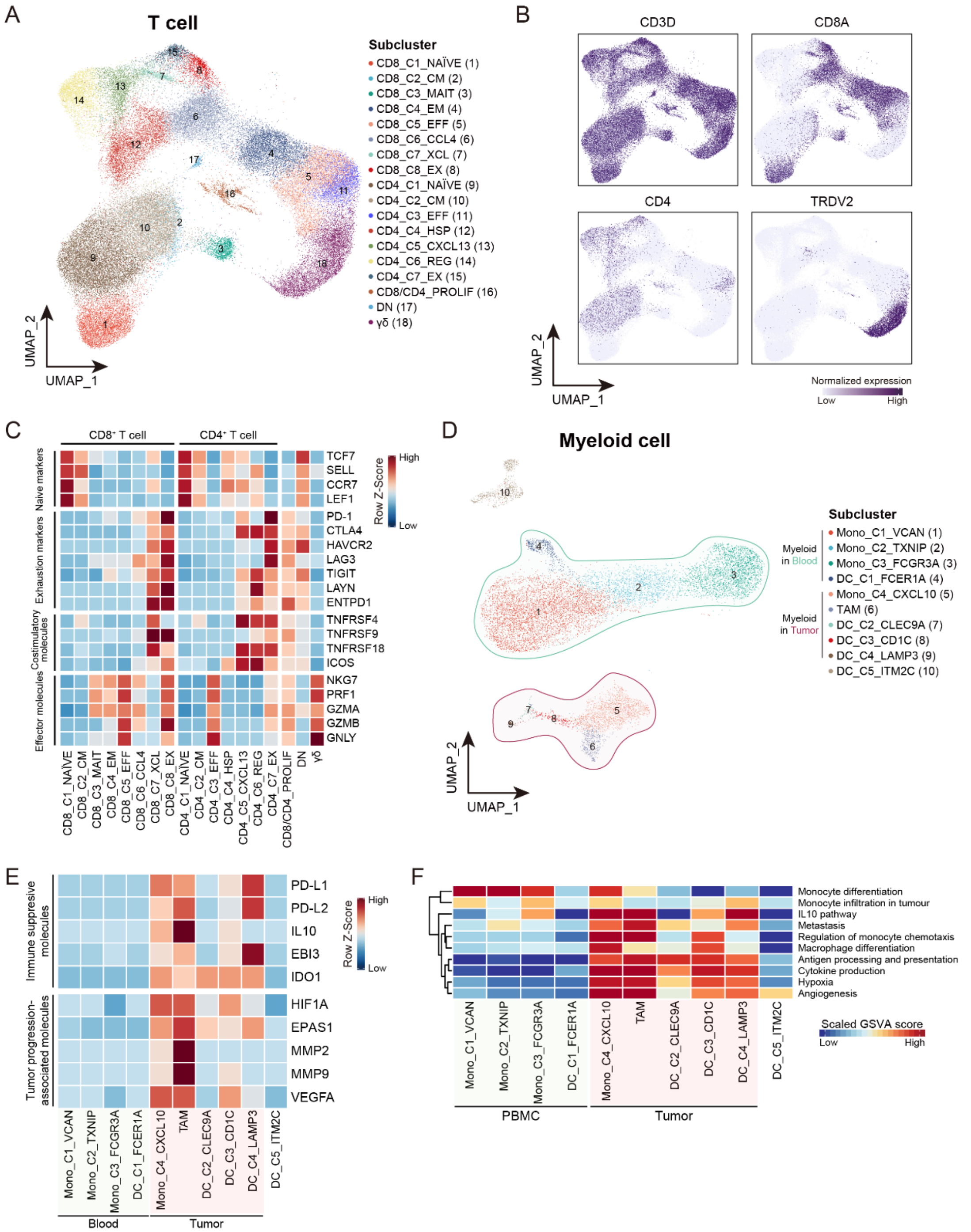

Figure S5 | Expression profiles of T and myeloid cell clusters in NKTCL.

A. UMAP plot showing 70,175 T cells grouped into 18 cell clusters, which were annotated based on their unique expression of canonical markers (**Table S3b**), including naïve (CD8_C1_NAÏVE and CD4_C1_NAÏVE), central memory (CD8_C2_CM and CD4_C2_CM), MAIT (CD8_C3_MAIT), effector memory (CD8_C4_EM), effector (CD8_C5_EFF and CD4_C3_EFF), tissue-resident memory (CD8_C6_CCL4, CD8_C7_XCL, and CD4_C4_HSP), Th1-like (CD4_C5_CXCL13), regulatory

(CD4_C6_REG), exhausted (CD8_C8_EX and CD4_C7_EX), high-proliferative (CD8/CD4_PROLIF), double negative (DN), and γδ T cells. Each dot represents a cell, colored according to its cell cluster as indicated at the right panel.

B. UMAP plots showing the normalized expression of markers genes (*CD3D*, *CD8A*, *CD4*, and *TRDV2*). Each dot represents a cell, and the depth of color from light grey to deep purple represents low to high expression.

C. Heatmap showing the expression levels of signature genes (rows) for T cell clusters (columns). Filled colors from blue to red in the squares represent normalized expression levels from low to high as scaled in row direction (row Z-score).

D. UMAP plot showing 14,330 myeloid cells grouped into 10 cell clusters, including four and five clusters mainly restricted in peripheral blood and tumor, respectively. Myeloid cells were defined as four clusters of monocytes, one cluster of tumor-associated macrophages (TAMs), and five clusters of dendritic cells (DCs) based on the expression of well-known marker genes (**Table S3c**). Each dot represents a cell, colored according to its cell cluster as indicated at the right panel.

E. Heatmap showing the expression levels of selected genes (rows) for myeloid cell clusters (columns). Filled colors from blue to red in the squares represent normalized expression levels from low to high as scaled in row direction (row Z-score). Upregulation of immunosuppressive genes (like PD-L1, PD-L2, IL10, EBI3, and IDO1) and highest expression of *HIF1A*, *EPAS1*, *MMP2*, *MMP9*, and *VEGFA* were observed in TAMs.

F. Heatmap showing activations of selected signaling pathways (rows) among myeloid cell clusters (columns). Filled colors from blue to red in the rectangles represent GSVA scores from low to high. Enrichment of cancer hallmarks including hypoxia, metastasis, and angiogenesis was observed in TAMs.

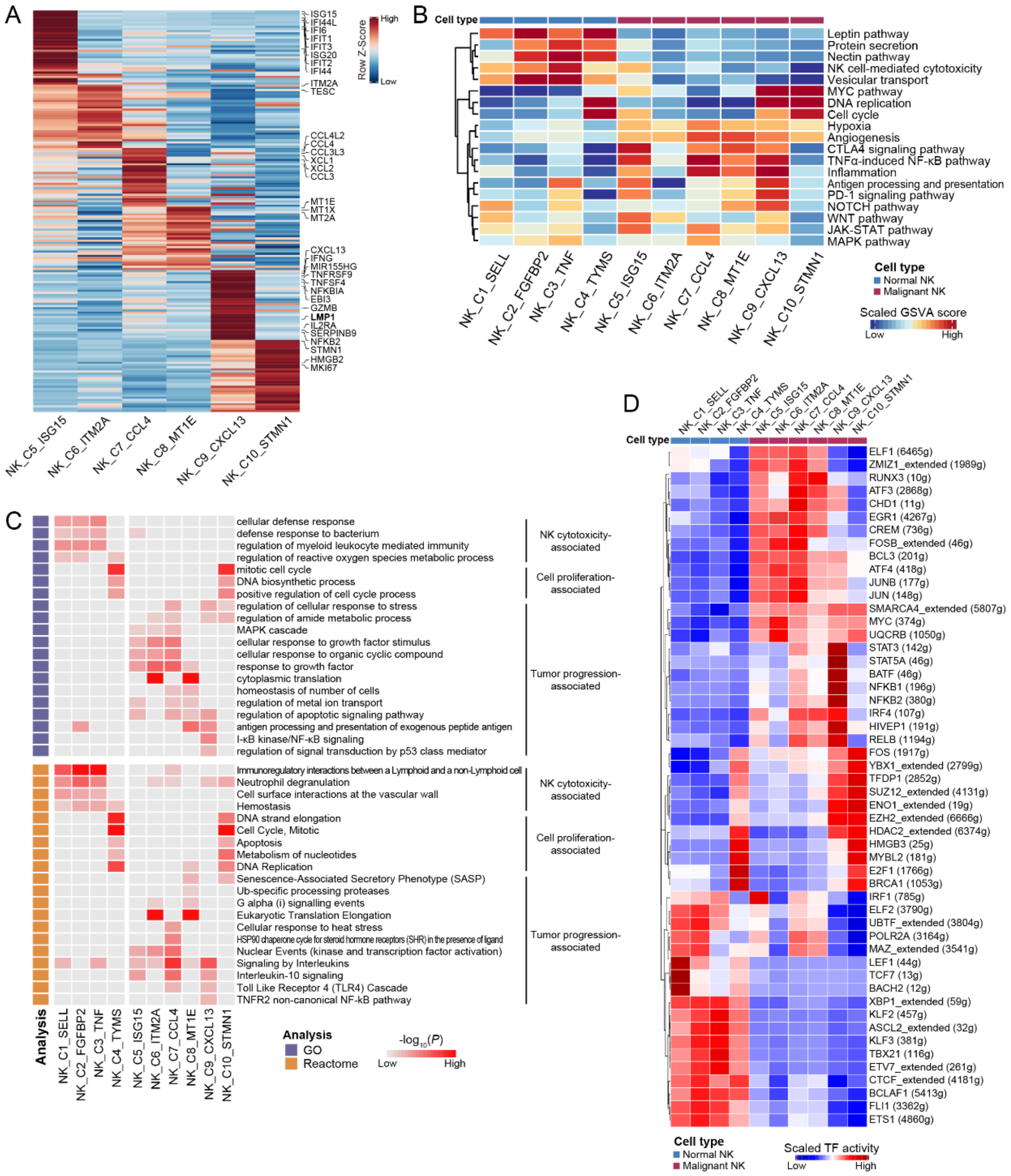

Figure S6 | Identification of heterogeneous malignant NK cell clusters.

A. Heatmap showing the expression levels of top differentially expressed genes (rows) for six malignant NK clusters (columns). Filled colors from blue to red in lines represent normalized expression levels from low to high as scaled in row direction (row Z-score).

B-D. Heatmaps showing the GSVA scores (B) or *P* values (C) of signaling pathways (rows) and SCENIC activity of transcription factors (rows; D) among normal and malignant NK cell clusters (columns). NK clusters of either normal (blue) or malignant NK (purple) are indicated as rectangles on top. Filled colors from blue to red (B and D) or from light grey to red (C) in the heatmap boxes represent GSVA scores (B), scaled *P* values (C),

and SCENIC activity (D) from low to high.

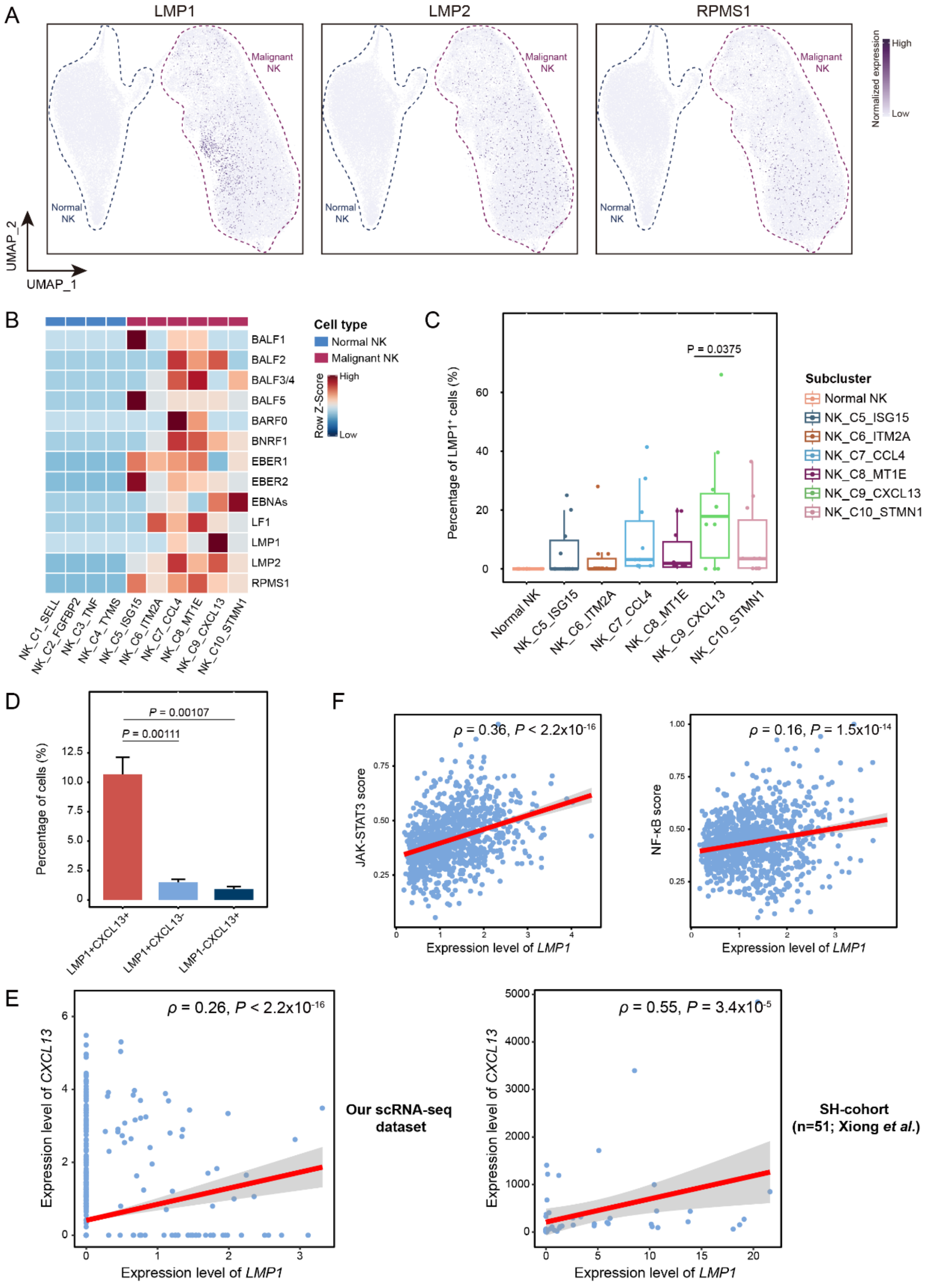

Figure S7 | Expression of EBV-encoded genes and activation of oncogenic signaling pathways in malignant NK cells.

A. UMAP plots related to **Figure 2A** showing the normalized expression of EBV encoded-genes, *LMP1*, *LMP2*, and *RPMS1* among normal and malignant NK cells. Each dot represents a cell, and the depth of color from light grey to deep purple represents low to high expression.

B. Heatmap showing the expression levels of 13 EBV-encoded genes (rows) for each NK cell cluster (columns). NK clusters of either normal (blue) or malignant NK (purple) are indicated as rectangles on top. Filled colors from blue to red in the squares represent normalized expression levels from low to high as scaled in row direction (row Z-score).

C. Box plots showing the proportions of $LMP1^+$ cells for normal NK cells and each malignant NK cluster in NKTCL patients. Center lines denote median values; whiskers denote 1.5× the interquartile range; colored dots denote the proportion of $LMP1^+$ cells for each patient in each NK cell type. Comparison was made using Wilcoxon rank-sum test.

D. Bar plot showing the percentages of $LMP1^+CXCL13^+$ double-positive cells and $LMP1^+/CXCL13^+$ single-positive cells from the results of IF staining assays (n=6; **Figure 2B**). Comparisons were made using paired Student's t-test.

E. Scatter plots showing the correlations between the expression levels of *LMP1* and *CXCL13*. Each dot represents a malignant NK cell from our scRNA-seq cohort (left panel) or a tumor sample from the SH-cohort (n=51; right panel), with its corresponding expression levels of two genes indicated at the x- and y-axis, respectively.

F. Scatter plots showing the expression of *LMP1* significantly correlated to the activation of JAK-STAT3 (left panel) and NF-κB signaling pathways (right panel) in malignant NK cells from our scRNA-seq cohort. Each dot represents a cell, with its corresponding expression level of *LMP1* indicated at the x-axis, and the module scores of JAK-STAT3 and NF-κB signaling pathways (**Table S7**) indicated at the y-axis.

Correlations were assessed using Spearman's rank correlation test.

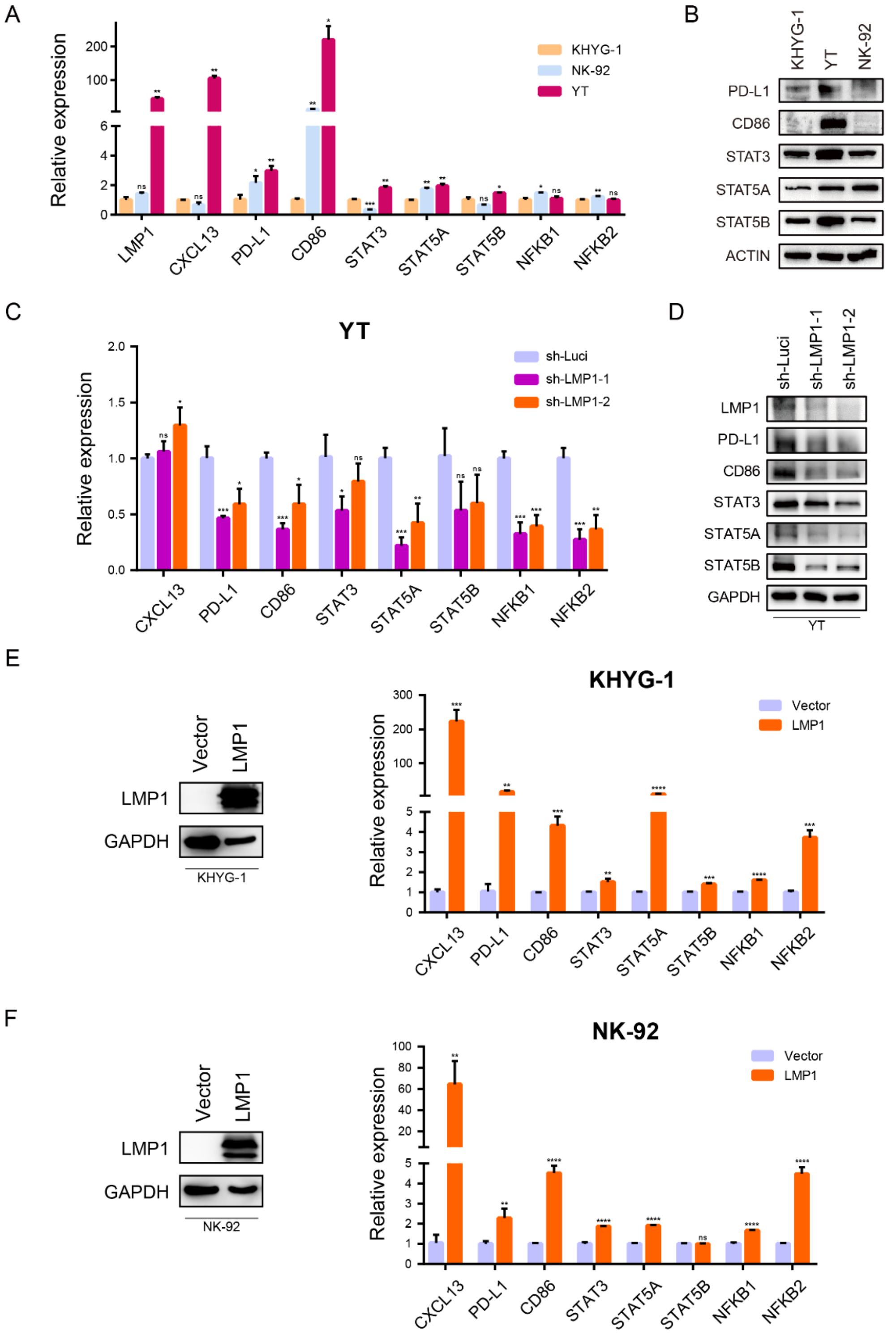

Figure S8 | Expression of NK_C9_CXCL13 signature genes in NKTCL cell lines in response to LMP1 expression.

A. Bar plot showing the relative expression levels (y-axis) of signature genes (x-axis) in NKTCL cell lines (KHYG-1, NK-92, and YT) as indicated with corresponding colors, including the marker genes of NK_C9_CXCL13 (*LMP1* and *CXCL13*) as well as

oncogenic molecules (*STAT3*, *STAT5A*, *STAT5B*, *NFKB1*, and *NFKB2*) and immune checkpoint molecules (*PD-L1* and *CD86*) notably expressed in NK_C9_CXCL13 cells.

B. Western blotting assay showing the protein expression of abovementioned signature genes for NK_C9_CXCL13 cell in NKTCL cell lines with ACTIN as control.

C-D. Bar plot (C) showing the relative expression levels (y-axis) of signature genes (x-axis) in $LMP1^+$ NKTCL cell line (YT) with LMP1 knockdown (sh-LMP1-1 and sh-LMP1-2) or control (sh-Luci), and western blotting assay (D) showing the expression of indicated proteins in the YT cell lines in (C).

E-F. Western blotting assays showing the protein expression of LMP1 and GAPDH in $LMP1^-$ NKTCL cell lines KHYG-1 (E) and NK-92 (F) infected with lentivirus-expressing *LMP1* or empty vector control (left panels), and bar plots showing the relative expression levels (y-axis) of signature genes (x-axis) with or without LMP1 overexpression (right panels).

Comparisons were made using Student's t-test, and results of bar plots are shown as mean value ± standard deviation (SD). $^{ns}P \geq 0.05$, $^{*}P < 0.05$, $^{**}P < 0.01$, $^{***}P < 0.001$, $^{****}P < 0.0001$.

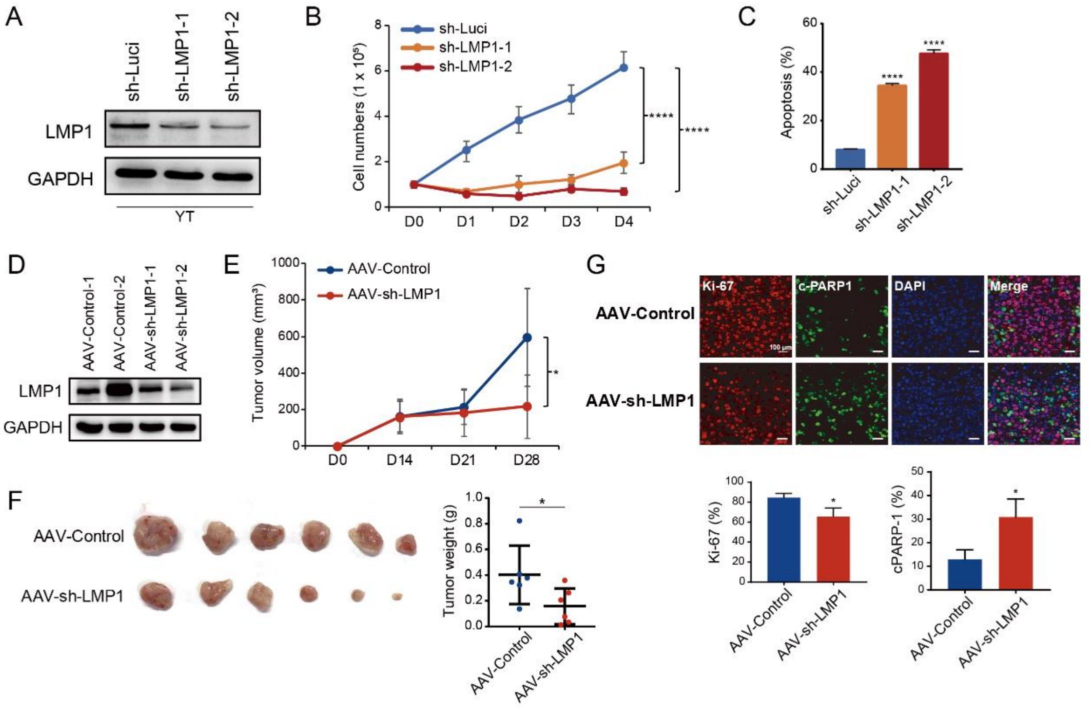

Figure S9 | *In vitro* and *in vivo* effects of LMP1 knockdown in NKTCL cells.

Stable YT cell lines were established with the knockdown of LMP1 mediated by either shRNAs (sh-LMP1-1/-2) or AAV (AAV-sh-LMP1) or with the respective control vectors (sh-Luci or AAV-control).

A. Western blotting assay reveals the protein expression of LMP1 and GAPDH in YT cells infected with sh-LMP1-1, sh-LMP1-2, or sh-Luci control.

B. Cell growth curves indicate the cell numbers of these cells at each time point (D, day).

C. Bar plot indicates the percentages of apoptotic cells according to their staining with Annexin V using fluorescence-activated cell sorting (FACS).

D. Western blotting assay reveals the protein expression of LMP1 and GAPDH in the xenografted tumors derived from YT cells with AAV-expressing shRNAs.

E. Tumor growth of the xenografts with YT cells infected with AAV as indicated at different time courses (D, day).

F. Tumor size (left panel) and tumor weight (right panel) for the xenografts excised from (E).

G. Multiplex IF staining assays (top panel) for the protein expression of Ki-67 and c-PARP1 in malignant NK cells for the tumor section of the xenografts excised from (E) and bar plots (bottom panel) for the percentages of Ki-67^{+}/c-PARP1^{+} cells.

Comparisons were made using Student's t-test, and results for growth curves and bar plots are shown as mean value ± standard deviation (SD). $^{ns}P \geq 0.05$, $^{*}P < 0.05$, $^{**}P < 0.01$, $^{***}P < 0.001$, $^{****}P < 0.0001$. Scale bars, 100 μm.

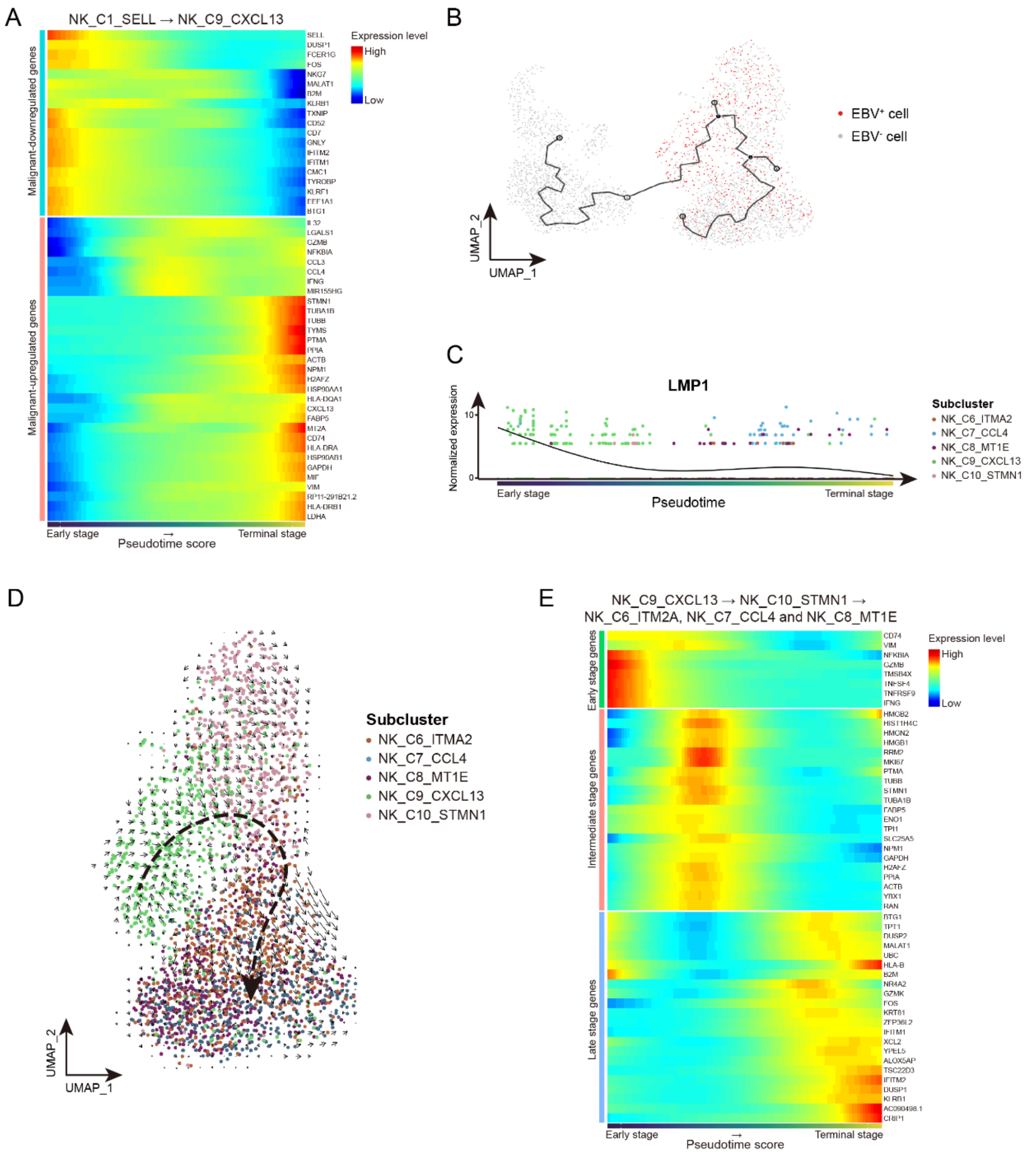

Figure S10 | Pseudotime trajectory analysis of malignant and normal NK cells, related to Figure 2H.

A. Heatmap showing the expression levels of downregulated and upregulated genes (rows) with significant difference from normal naïve NK cells (NK_C1_SELL) to LMP1⁺ malignant NK cells (NK_C9_CXCL13). Filled colors from blue to red in vertical lines represent normalized expression levels from low to high in each cell.

B. Pseudotime development trajectory of NK cells. Each dot represents a cell in trajectory projection, colored according to whether it expresses EBV-encoded genes (red) or not (grey).

C. Scatter plot showing the normalized expression levels (y-axis) of *LMP1* along the

pseudotime trajectory of malignant development (x-axis). Each dot represents a cell, colored according to its cell cluster as indicated at the right panel. The inner line represents the trend of *LMP1* expression with increasing pseudotime scores.

D. UMAP plot showing the estimated RNA velocity for malignant NK cells. Each dot represents a cell, colored according to its cell cluster as indicated at the right panel; each small arrow indicates the developmental direction for nearby cells; the dashed line represents the overall developmental direction of malignant NK cells.

E. Heatmap showing the expression levels of downregulated and upregulated genes (rows) with significant difference from NK_C9_CXCL13 (early stage) to NK_C10_STMN1 (intermediate stage) and subsequently to NK_C6_ITM2A, NK_C7_CCL4, and NK_C8_MT1E (late stage). Filled colors from blue to red in vertical lines represent normalized expression levels from low to high in each cell.

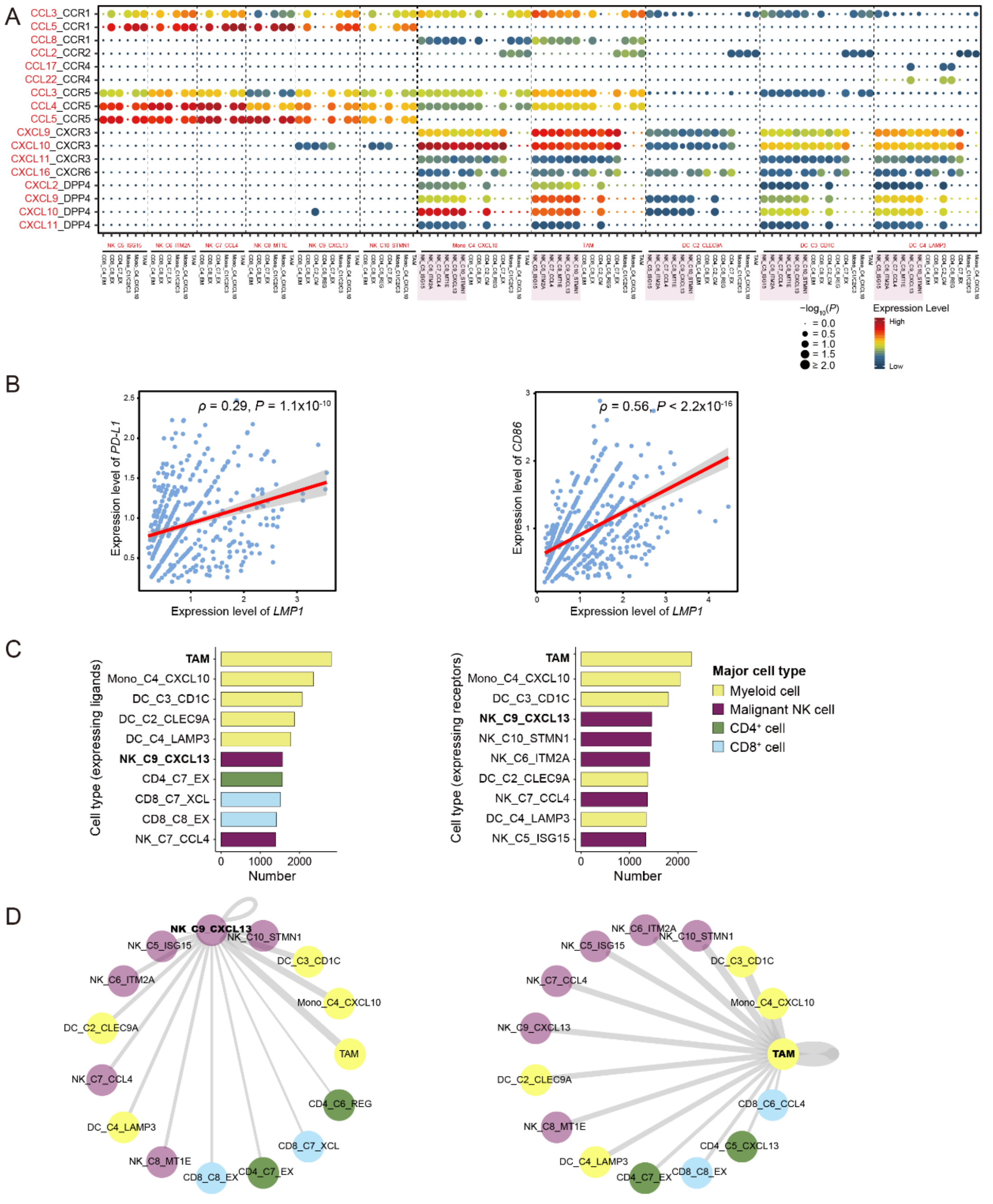

Figure S11 | Chemotactic and immunosuppressive characteristics of NKTCL TME.

A. Dot plot showing ligand-receptor interactions (rows) of chemokines with significant difference between selected cell clusters (columns; the major left and right divisions annotated by the vertical dashed line in bold for malignant NK and myeloid cells, respectively). Ligands (red at the left; row) expressed by source cells (red at the bottom; column) are identified to interact with receptors (black at the left; row) expressed on target cells (black at the bottom; column). The highlighted color modules at the bottom represent the target cells of either malignant NK cells (light purple) or immune cells

(white). *P* values estimated using one-sided permutation test (-log10 scaled) are indicated by circle sizes and the means of the average expression levels of two interacting molecules are indicated by filled colors, with blue to red representing low to high expression.

B. Scatter plots showing the expression of *LMP1* significantly correlated with that of *PD-L1* (left panel) and *CD86* (right panel) in malignant NK cells. Each dot represents a cell, with its corresponding expression level of *LMP1* indicated at the x-axis, and the expression levels of *PD-L1* and *CD86* indicated at the y-axis. Correlations were assessed using Spearman's rank correlation test.

C. Bar plots showing top 10 cell clusters with the highest expression of ligands (left panel) and receptors (right panel), colored according to their major cell types as indicated at the right panel. The numbers of ligands/receptors and the top 10 cell clusters are indicated at the x- and y-axis, respectively.

D. Cellular interaction networks of top 15 cell clusters with the most intensive interactions with NK_C9_CXCL13 (left panel) and TAM (right panel). NK_C9_CXCL13 cells or TAMs were treated as source cells expressing ligands. The thickness of each line indicates the number of ligand-receptor pairs in scale estimated between the corresponding two cell types, and the circles are colored according to the major cell types.

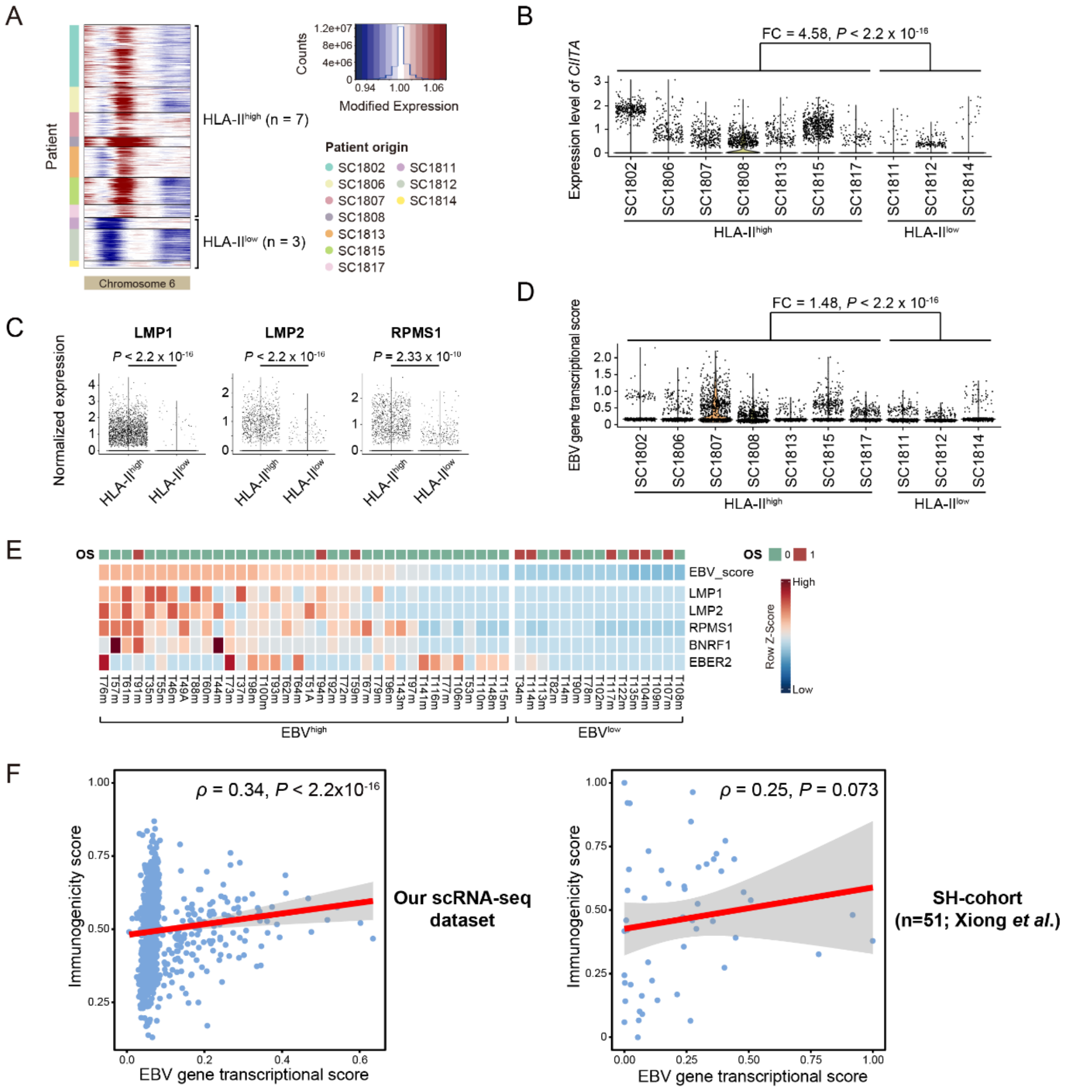

Figure S12 | Characterization of EBV^{high} and EBV^{low} patients with NKTCL.

A. Heatmap showing the large-scale CNVs within chromosome 6 region in malignant NK cells for each patient (rows; colored according to sample id as indicated at the right panel) related to **Figure 6A**. CNVs are inferred as gains (red) or losses (loss) according to the average expression of 100 genes within chromosome 6.

B. Violin plot showing the normalized expression of *CIITA* in malignant NK cells for each patient. Patients of two groups and the expression levels of *CIITA* are indicated at the x- and y-axis, respectively.

C. Violin plots showing the normalized expression of EBV-encoded *LMP1*, *LMP2*, and *RPMS1* in malignant NK cells for the HLA-II^{high} and HLA-II^{low} patients. Patients of two groups and the expression levels of genes are indicated at the x- and y-axis, respectively.

D. Violin plot showing the transcriptional scores of EBV-encoded genes in malignant NK cells for each patient. Patients of two groups and their module scores of EBV gene transcription are indicated at the x- and y-axis, respectively.
E. Heatmap showing the expression levels of EBV-encoded genes (rows) for NKTCL samples of the SH-cohort (n=51; columns). Filled colors in the rectangles from blue to red represent normalized expression levels from low to high as scaled in row direction (row Z-score). Corresponding clinical information of death (dark red) or not (cyan) is indicated as squares on top.
F. Scatter plots showing the correlations between the module scores of EBV gene transcription and immunogenicity. Each dot represents a malignant NK cell from our scRNA-seq cohort (left panel) or a tumor sample from the SH-cohort (n=51; right panel), with its corresponding module scores of EBV gene transcription and immunogenicity (**Table S7**) indicated at the x- and y-axis, respectively. Correlations were assessed using Spearman's rank correlation test.

Comparisons between two groups were made using Wilcoxon rank-sum test.

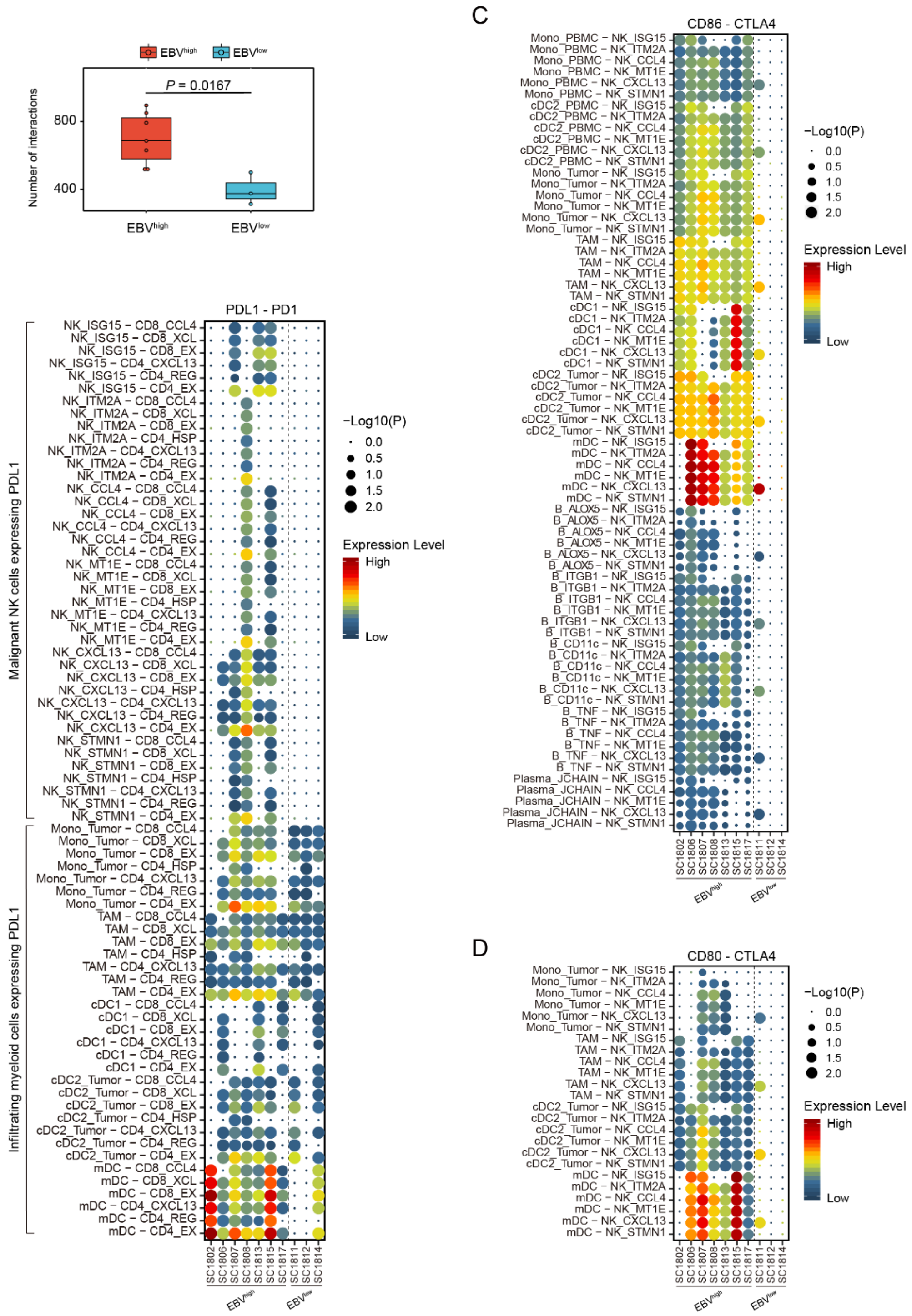

Figure S13 | Intensive immunosuppressive interactions of malignant NK cells in EBV^{high} samples.

A. Box plot showing the numbers of cellular interactions between malignant NK cells and immune cells (y-axis) in patient groups of EBV^{high} or EBV^{low}. Center lines denote median values; whiskers denote 1.5× the interquartile range; colored dots denote the numbers of interaction involved in malignant NK cells for each patient. Comparison

was made using Wilcoxon rank-sum test.

B-D. Dot plots showing the interactions of PDL1-PD1 (B), CD86-CTLA4 (C), and CD80-CTLA4 (D) among malignant and immune cells for each patient (columns). The cell-cell interaction pairs of source cells and target cells are indicated at rows. *P* values estimated using one-sided permutation test (-log10 scaled) are indicated by circle sizes and the means of the average expression levels of two interacting molecules are indicated by filled colors, with blue to red representing low to high expression (right).

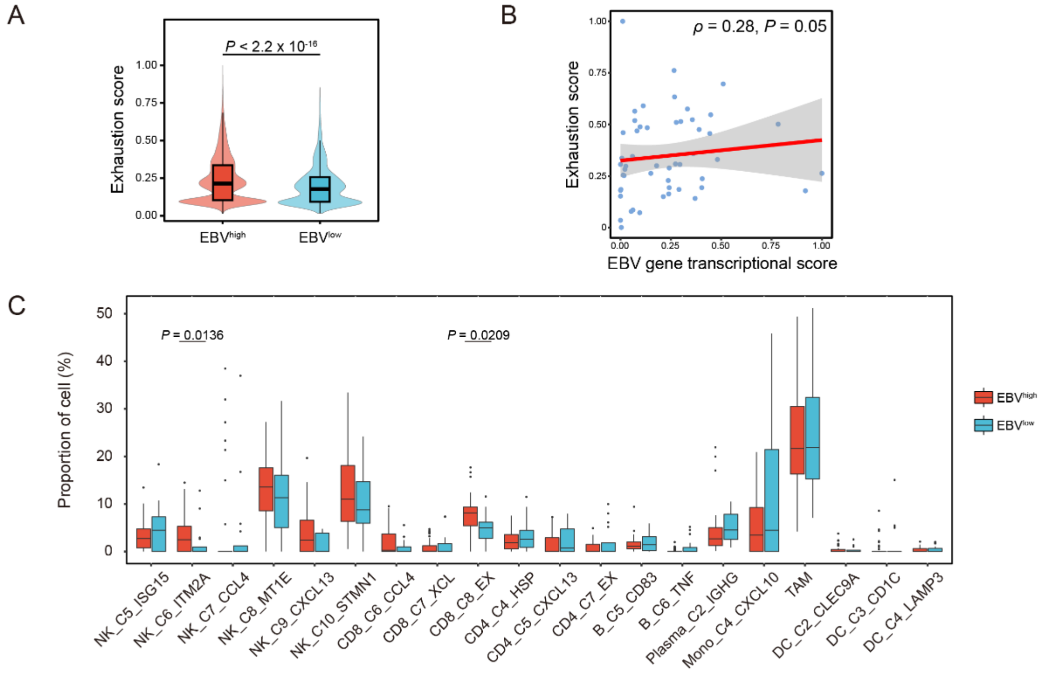

Figure S14 | Enhanced immunosuppressive property of malignant NK cells in EBV^{high} samples.

A. Violin plot showing the exhaustion scores of tumor-infiltrating T cells for EBV^{high} and EBV^{low} patients. Patients of two groups and exhaustion module scores (**Table S7**) are indicated at the x- and y-axis, respectively.

B. Scatter plot showing the significant correlation between the module scores of EBV gene transcription and immune exhaustion in NKTCL samples from the SH-cohort (n=51). Each dot represents a tumor sample, with its corresponding module scores of EBV gene transcription and immune exhaustion (**Table S7**) indicated at the x- and y-

axis, respectively. Correlation was assessed using Spearman’s rank correlation test.

C. Box plot showing the fractions (y-axis) for each cell population (x-axis) in patient groups of EBV^{high} (red) or EBV^{low} (blue) from the SH-cohort. Center lines denote median values; whiskers denote 1.5× the interquartile range; colored dots denote the proportion of each cell type in each patient.

Comparisons between two groups were made using Wilcoxon rank-sum test.

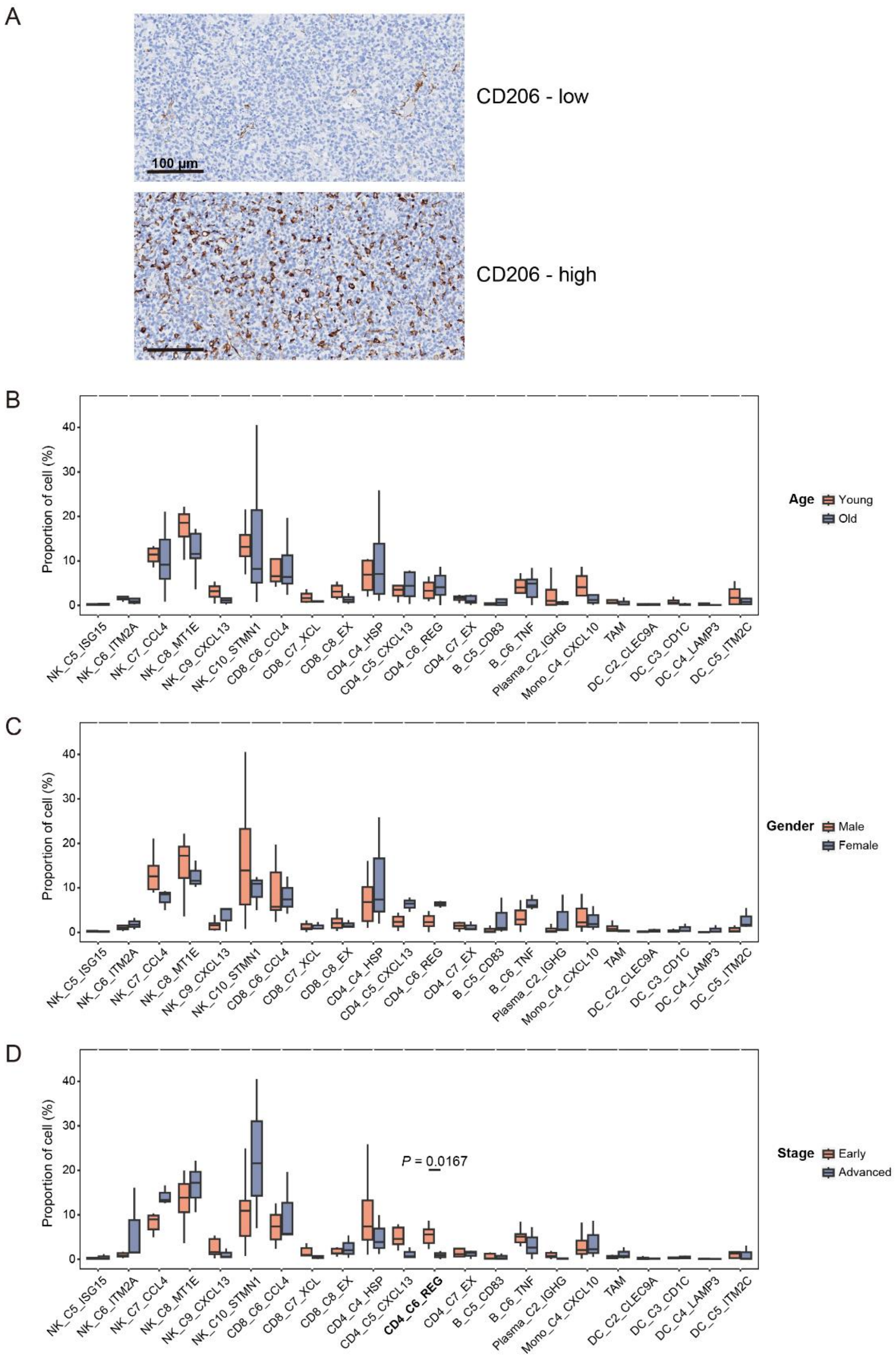

Figure S15 | Clinical associations with TME components in NKTCL.

A. Representative immunohistochemical staining images of low (top panel) and high protein expression (bottom panel) of CD206 in formalin-fixed paraffin-embedded (FFPE) NKTCL tissue biopsies from the SC-cohort (n=55). Scale bars, 100 μm.

B-D. Box plots showing the fractions (y-axis) for each cell population (x-axis) in patient groups categorized according to their clinical characteristics, including age (B), gender

(C), and tumor stage (D), from our scRNA-seq cohort. Center lines denote median values; whiskers denote 1.5× the interquartile range; colored dots denote the proportion of each cell type in each patient. Comparison was made using Wilcoxon rank-sum test.
